# Spatiotemporal analysis and predicting rainfall trends in a tropical monsoon-dominated country using MAKESENS and machine learning techniques

**DOI:** 10.1038/s41598-023-41132-2

**Published:** 2023-08-25

**Authors:** Md. Moniruzzaman Monir, Md. Rokonuzzaman, Subaran Chandra Sarker, Edris Alam, Md. Kamrul Islam, Abu Reza Md. Towfiqul Islam

**Affiliations:** 1 Department of Geography and Environmental Science, Begum Rokeya University, Rangpur, Bangladesh; 2 Faculty of Resilience, Rabdan Academy, 22401 Abu Dhabi, United Arab Emirates; 3 Department of Geography and Environmental Studies, University of Chittagong, Chittagong, 4331 Bangladesh; 4 Department of Civil and Environmental Engineering, College of Engineering, King Faisal University, 31982 Al Hofuf, AlAhsa Saudi Arabia; 5 Department of Disaster Management, Begum Rokeya University, Rangpur, 5400 Bangladesh; 6 Department of Development Studies, Daffodil International University, Dhaka, 1216 Bangladesh

**Keywords:** Climate sciences, Hydrology

## Abstract

Spatiotemporal rainfall trend analysis as an indicator of climatic change provides critical information for improved water resource planning. However, the spatiotemporal changing behavior of rainfall is much less understood in a tropical monsoon-dominated country like Bangladesh. To this end, this research aims to analyze spatiotemporal variations in rainfall for the period 1980–2020 over Bangladesh at seasonal and monthly scales using MAKESENS, the Pettitt test, and innovative trend analysis. Multilayer Perception (MLP) neural network was used to predict the next 8 years' rainfall changes nationally in Bangladesh. To investigate the spatial pattern of rainfall trends, the inverse distance weighting model was adopted within the ArcGIS environment. Results show that mean annual rainfall is 2432.6 mm, of which 57.6% was recorded from July to August. The Mann–Kendall trend test reveals that 77% of stations are declining, and 23% have a rising trend in the monthly rainfall. More than 80% of stations face a declining trend from November to March and August. There is a declining trend for seasonal rainfall at 82% of stations during the pre-monsoon, 75% during the monsoon, and 100% during the post-monsoon. A significant decline trend was identified in the north-center during the pre-monsoon, the northern part during the monsoon, and the southern and northwestern portions during the post-monsoon season. Predicted rainfall by MLP till 2030 suggests that there will be little rain from November to February, and the maximum fluctuating rainfall will occur in 2025 and 2027–2029. The ECMWF ERA5 reanalysis data findings suggested that changing rainfall patterns in Bangladesh may have been driven by rising or reducing convective precipitation rates, low cloud cover, and inadequate vertically integrated moisture divergence. Given the shortage of water resources and the anticipated rise in water demand, the study's findings have some implications for managing water resources in Bangladesh.

## Introduction

Bangladesh is a tropical monsoon-dominated nation with a humid climate characterized by substantial monsoon rainfall and less rainfall during the rest of the year. This country is primarily an agrarian economy, with around 80% (145 million) of its people involved in various agricultural pursuits, directly or indirectly^1–3^. As a result, the effects of climatic fluctuation and change on the country's economic expansion and food security are considerable^4,5^. Rainfall has great significance as a hydrologic parameter, is a vital feature of climate change, and significantly affects food production, harvesting, human resources, and the natural environment^6^. Relevant and essential rainfall information, such as daily, weekly, and monthly assessments, are required to maintain an adequate water management system^7,8^. The historical rainfall trend analysis can make predictions^9^. Rainfall predicting is a serious concern that has great importance in government and scientific research, plays a significant role in protecting human lives and resources^10^; and also contributes to water resource management, the production of hydroelectricity, warning of floods and droughts, and urban sewerage systems^11,12^.

Since the 1950s, the global climate system has seen unprecedented changes, and it is now undeniable that global warming impacts climate^13^. There are many different temporal and geographical scales at which precipitation variability as a component of climate change occurs. Worldwide studies have shown increasing rainfall variability on a global scale^14,15^, and the Indian Sub-continent, including India^16^ and Pakistan^17,18^. Many studies^19–21^ have examined the spatiotemporal patterns of rainfall in climatic zones across the world^22–25^. Some research has been conducted on the semi-regional scale of rainfall variability^26–29^. Throughout the past 100 years, the amount of rainfall on Earth has increased by around 2%^14^, but according to IPCC^30^, its growth has always been unequal in terms of time and space. Increases in rainfall have been found, in particular, in the Middle East, Central Asia^31^, Brazil region, the eastern part of America and Canada, and Northern Europe^32,33^, and Australia^34^. Ren et al. ^35^ observed for the annual average rainfall over the Tibetan Plateau in China, similar trends have been seen during the previous few decades, also the same pattern in India^33,36^, in northwest Pakistan^17,18^, and in the China-Pakistan Economic Corridor area^37^. However, the Yellow River Basin, China^38^, the Mediterranean region^29^, and Iran have all seen a decline in annual mean rainfall^19^. A significant upward trend in annual, monsoon, and pre-monsoon rainfalls has lately been found in Bangladesh, according to a growing number of earlier research^39–41^. In the past several decades, additional investigations have declined in the western and northwestregions^7,42^. Moreover, Hossain et al.^43^ noted an increasing trend in the mean annual rainfall in the southern coastline area of Bangladesh. Rahman and Islam^44^ assert a great deal of regional and temporal variation in rainfall. By 2100, monsoon rainfall will rise by 12%, while winter rainfall will reduce by 10% in Bangladesh, according to the Water Resources Planning Organization^45,46^. Moreover, Basak et al.^47^ predict a yield decline of between 3.3 and 24.2% and a 10 mm decrease in winter rainfall. According to certain studies, Bangladesh's seasons might see long-term variations in rainfall^7,48,49^.

Recent years have seen several studies done on rainfall trends around the world^17,26,29,31,50–52^, and in Bangladesh^40,42,43,53,54^. Most of the studies used the Simple linear trend^55^, the Mann–Kendall test^33,40^, the Mann–Kendall and Spearman’s rho tests^17,46^, Spearman’s rank test^23^, Pre-Whitening approach^57^ to detect the rainfall trend. However, combining the Mann–Kendall test and Sen’s Slope estimator (MAKESENS) technique is best for detecting rainfall trends^27,58,59^. The present study used the Mann–Kendall test and Sen’s slope estimator technique to identify the trend in rainfall. The previous studies focused on several parts of Bangladesh, such as the northern part ^42^, the South-West coast^43,53^, eastern Sylhet region^56^, but this present study was conducted over Bangladesh, exploring rainfall data from 28 weather stations. Previous studies examined fewer weather station data (for example, 12 weather station data used by Endo et al.^40^, 15 weather stations by Rahman et al. in 2017^46^, and 17 weather stations by Shahid in 2010 ^60^. Few recent types of research have utilized various time frames for various stations^54^. The previous studies analyzed the rainfall trend for some parts of the year, including the monsoon season^39,55,61^, the pre-monsoon and monsoon seasons^40^, and the pre-monsoon and post-monsoon seasons^62^. However, this study examined yearly rainfall trends with pre-monsoon, monsoon, and post-monsoon seasons. Most previous studies were conducted on annual and seasonalscales^26,50^. Few studies have analyzed monthly rainfall trends^23^. However, in Bangladesh, no comprehensive study focuses on monthly rainfall. Also, no previous studies have assessed spatiotemporal rainfall trends in Bangladesh like other studies worldwide^36,63^. To fill this gap, this present study spatiotemporally examined rainfall trends on a monthly and seasonal scale. The majority of previous research on rainfall predicting has used Linear Regression^64^, Adaptive Neuro-Fuzzy Inference System (ANFIS)^65^, Genetic Algorithm (GA)^66^, Mann Kendall test, Deep Learning Approach, Feed Forward Neural Network (FFNN), Empirical and Dynamical Methods, and Autoregressive Integrated Moving Average (ARIMA)^10,11,46,67–70^. Some innovative strategies have been widely used in recent years: wavelet transforms, couple-wavelet neural networks^71^, genetic algorithms, and uncertainty analysis for rainfall prediction^9^. Different studies focus on rainfall prediction in Bangladesh using different machine learning models as ARIMA^46,72^, Weather Research and Forecast (WRF) model^73^, regression analysis^74^. These studies are focused on seasonal rainfall, not monthly rainfall. Rainfall usually fluctuates yearly and at various times due to seasonal variations. In time series research, the rainfall amount graph shows fluctuations^13^. Predicting rainfall is challenging because of rainfall's multidimensional and nonlinear characteristics^6,10,11^. These approaches predict rain based on its trend nature. Previous studies predict rainfall for 10–15 years based on time series rainfall data^27,70^. Artificial Neural Networks (ANN) is an inverse traditional meteorological prediction method based on self-adaptive mechanisms^75^. ANN can predict rainfall with its fluctuated nature^76^. For a powerful weather predict purpose, artificial neural networks are employed ^77^. Though few studies used ANN techniques for predicting rainfall, they focus on only seasonal rainfall^11,27,78^. Banik et al.^11^ used the ANN technique for predicting rainfall in Bangladesh, but only for monsoon rainfall. The multilayer perceptron (MLP), trained using the BP technique, is the most often used ANN design in hydrologic modelling^139^. To fill this gap, the present study used the multi-layer perception (MLP) model of the ANN technique to predict monthly and seasonal (pre-monsoon, monsoon, and post-monsoon) rainfall in Bangladesh. ANN's MLP model can handle both low peak and high rainfall values as well as extremely non-linear rainfall data^79,80^. The use of MLP-based ANN for rainfall prediction yields consistent results^10,81–83^. For more than 48 inputs, the MLP model produces more accurate results^84,85^. The MLP model of ANN is widely used for predicting rainfall for more than 15 years^27^.

Based on the aforementioned discussion, the peculiarities of Bangladesh's spatial distribution and temporal rainfall have not yet been the subject of any study. The fundamental limitation is insufficient coverage by meteorological stations or lack of a variety of data sources of the earlier research. Although several studies^7,49,62,86^ have previously analyzed the regional-level variations in rainfall in Bangladesh over the last decades, no extensive study has been carried out to identify the trend analysis across Bangladesh. For the various regions of Bangladesh, the earlier research solely considered the MK test and change point detection methods employing Pettitt's test. This research also considers a thorough statistical investigation of climate change from the perspective of rainfall patterns. Historical rainfall trend analysis and predicting are critical in many disciplines, including water resource and ecosystem management, and sustainable agriculture planning^52^. However, the spatiotemporal shifting behavior of rainfall is considerably less accessible in Bangladesh as a tropical monsoon-dominated nation. The current study is, therefore, a step forward in terms of the sub-climatic zones, extended time frame, good data sources, handling missing datasets, quality control through outlier detection, autocorrelation identification and testing homogeneity, and innovative trend analysis with change point detection. This study focused on investigating spatiotemporal analysis and predicting rainfall trends on a seasonal scale across Bangladesh to address this problem. Therefore, the present study aims (a) to identify the spatiotemporal trend in monthly, seasonal, and annual rainfall in Bangladesh using the MAKESENS; (b) to predict monthly and seasonal rainfall till 2030 in Bangladesh using the MLP neural network; (c) to explore the causes of changes in rainfall pattern over Bangladesh. The uniqueness of our study is that it is the first to be conducted in Bangladesh, where we analyzed the spatiotemporal trends and predict in monthly and seasonal (pre-monsoon, monsoon, and post-monsoon) rainfall for a maximum (of 28) weather stations across the country. The prediction of future rainfall patterns for the country is the most distinctive feature of this research, and it will be highly beneficial for managing the country's water resources since rainfall is a crucial hydrological component.

## Materials and methods

### The study area's hydrological and geological background

**Figure 1:**
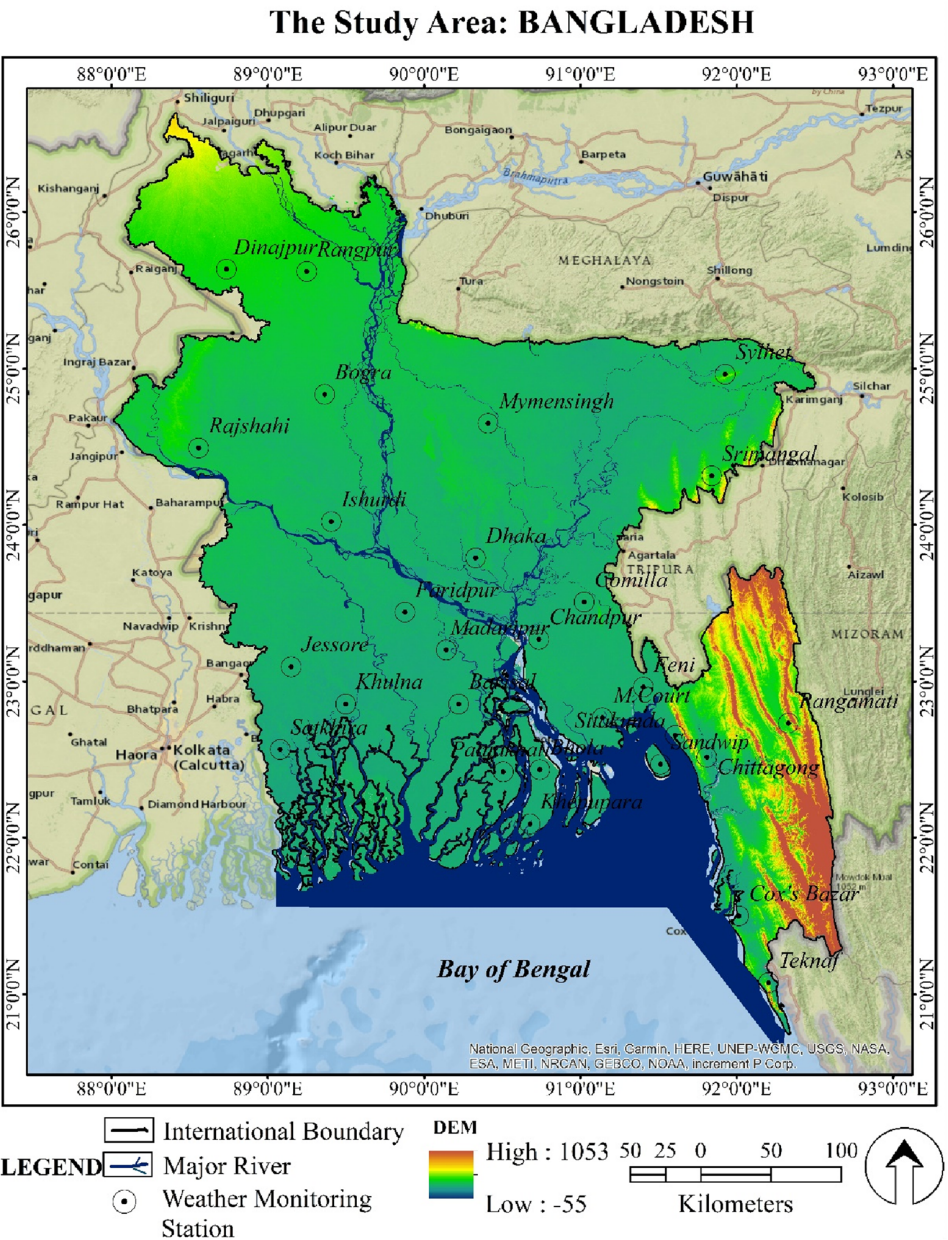
The location map of Bangladesh and the spatial distribution of the weather monitoring station, prepared by the authors using ArcGIS software version 10.5, (https://www.esri.com/en-us/arcgis/products); DEM data was derived from Shuttle Radar Topography Mission of USGS, (https://earthexplorer.usgs.gov/).

Bangladesh is situated in the northeastern region of South Asia, with its standard location lying from 20°34′ to 26°38′ N latitude and from 88°01′ to 92°41′ E longitude (Fig. 1). While the Bay of Bengal is located in the south, the spectacular Mountains can be seen to the north^87^.

Tropical cyclones and a humid, warm climate are common across Bangladesh, influenced by pre-monsoon, monsoon, and post-monsoon circulations^49^. Bangladesh's historical climate has had annual temperatures ranging from 15 to 34 °C, with an average temperature of roughly 26 °C. Bangladesh is a highly rainy nation, with an average annual rainfall of 2200 mm^88^.

### Sources of data and quality checks

We collect rainfall data (1981–2022) from the Bangladesh Meteorological Department (BMD) (http://live.bmd.gov.bd/bn/). Despite Bangladesh having 38 weather monitoring stations, we only consider the rainfall data from 28 of them as they consistently recorded rain over the study period. The selected weather monitoring stations are *Bhola*, *Barisal*, *Cox’s Bazar*, *Feni*, *Khepupara*, *M. Court*, *Patuakhali*, *Rangamati*, *Sandwip*, *Sitakunda*, *Chittagong*, *Teknaf*, *Sylhet*, *Srimongal*, *Rangpur*, *Dinajpur*, *Bogra*, *Ishurdi*, *Rajshahi*, *Jessore*, *Khulna*, *Satkhira*, *Comilla*, *Chandpur*, *Dhaka*, *Faridpur*, *Madaripur*, *Mymensingh* (Fig. 1). The maximum and minimum temperature, relative humidity, solar radiation, and wind speed data from 1981 to 2020 collect from the Bangladesh Agriculture Research Council (BARC) (http://www.barc.gov.bd/).

This study analyzes rainfall trends in Bangladesh on a monthly and seasonal scale. Bangladesh has three dominant seasons; these are (a) the Pre-monsoon (March–May), (b) the Monsoon (June–October), and (c) the Post-monsoon (November–February)^89^. The daily rainfall data have been converted to monthly mean rainfall data. Before assessing changes in rainfall, the data must undergo quality control since unreliable outliers might alter trend patterns^90^. The standard ratio method was used for estimating the missing rainfall data and clustered into three seasons. Multiple Imputation is used to handle outliers that have been detected. Using Durbin-Waston statistics, we examine autocorrelation in rainfall data, and the trend-free pre-whitening (TFPW) method was used to delete the correlation for the trend test. To accurately depict the natural fluctuations in rainfall, we test homogeneity before the trend test.

#### Missing data estimation

Using a typical ratio to balance the rainfall, the standard ratio approach^91^ calculates the missing data from neighboring stations. Multiple imputations (MI) are utilized for the missing numbers for which there is no accessible nearby station data. To obtain statistically accurate estimates for missing data, MI, a statistical procedure, comprises three phases^92^.

*Standard ratio approach:* the weighting factor in the following equation (Eq. 1) is determined by the ratios between the targeted station and nearby stations:

1 $$\mathrm{Y }=\frac{1}{n}\sum_{i-1}^{n}\frac{{N}_{s}}{{N}_{i}}{X}_{i}$$

where ${N}_{s}$ = average of the rainfall data currently available at the target station, ${N}_{i}$ = average of the rainfall data currently available at the *i*th neighboring station, and the total number of adjacent stations is n.

#### Outliers detection

The two-sided test's null hypothesis is that there are no outliers in the sample, whereas the alternate hypothesis is that either the lowest or highest number is an outlier. Grubbs' test^93^ is used to find outliers in the data.

*Grubbs’ test:* The time series data are ordered (${x}_{1}, {x}_{2}, {x}_{3}, \dots \dots ..{x}_{n}$) in ascending order, with ${x}_{1}$ denoting the lowest and ${x}_{n}$ is highest values of data points. The following t-statistic (Eq. 2) is used to check for outliers in the ${x}_{1}$ and ${x}_{n}$ points:

2 $${T}_{1} = \frac{\overline{x }-{x}_{1}}{s} \, \mathrm{and }{T}_{n}=\frac{{x}_{n} -\overline{x}}{s }$$

where ${T}_{1}$ is the lowest point t-statistics and ${T}_{n}$ for the highest points, X represents the time series' mean, while s represents its standard deviation. The observed value of Grubbs' statistic ${T}_{Obs}$ for a two-sided test is calculated using Eq. 3.

3 $${T}_{Obs}=\mathrm{max}\left({T}_{1}, {T}_{n}\right)$$

When ${T}_{Obs}$ > ${T}_{Crit}$, the Grubbs’ statistic ${T}_{Crit}$ is a critical value, and the null hypothesis is rejected significantly. In this study, the value is assumed to be 5%. The normalized z-scores^94,95^, are also computed and shown to visualize probable outliers and suspicious data. Any number beyond the predetermined range (− 1.96 and + 1.96) for a 5% significance level is considered suspicious, even if it might not be an outlier.

#### Autocorrelation identification

Autocorrelation in rainfall data was observed through the Durbin-Waston autocorrelation statistics.

*Durbin-Waston statistics:* Eq. 4 provides the commonly used formula for Durbin-Waston autocorrelation statistics^96^.

Durbin-Waston autocorrelation statistics

4 $$\mathrm{d}=\frac{\sum_{t=2}^{T}{({e}_{t}-{e}_{t-1})}^{2}}{\sum_{t=1}^{T}{e}_{t}^{2}}$$

If ${e}_{t}$ is the residual given by

$${e}_{t}={P}_{{e}_{t-1}}+ {V}_{t}$$

The experimental observation number, in this case, is T. The range of Durbin-Waston statistics values was 0–4. Only a value of 2 indicates the absence of positive or negative autocorrelation^96^. According to Ahmad et al.^17^, the correlation for trend tests was removed using the TFPW method.

#### Testing homogeneity

Climate data are inhomogeneous for measurement error, changes in the instrument's surrounding areas, and improper human handling^97^. Before trend analysis, the homogeneity must be checked; otherwise, the findings would show incorrect trends. In this current study, Alexandersson’s standard regular homogeneity test^98,99^ and Von Neumann’s ratio test^100^ were used for the homogeneity test.

*Alexandersson’s standard regular homogeneity test:* is used to look for abrupt shifts in the time series of meteorological and hydrologic information. According to the following equation (Eq. 5), Alexandersson^98^ suggests the statistic *T (k)* compares the mean of the first "*k*" years of the record with that of the latter "*n-k*" years:

5 $$T \left(k\right)= k{\overline{z} }_{1}^{2}+\left(n-k\right){\overline{z} }_{2}^{2} \,\text{for k }= 1, 2, 3, \dots ...,\mathrm{ n}$$

where “$\overline{X }$” = mean and “s” = standard deviation for an annual series ${X}_{i}$ (i is the year from 1 to n) with mean “$\overline{X }$” and “s”.

Again, ${\overline{z} }_{1}$ and ${\overline{z} }_{2}$ calculated from Eq. 6

6 $${\overline{z} }_{1}=\frac{1}{k}\sum_{i=1}^{k}\frac{({X}_{i}-\overline{X })}{s}\text{ and }{\overline{z} }_{2}=\frac{1}{(n-k)}\sum_{i=k+1}^{k}\frac{({X}_{i}-\overline{X })}{s}$$

If a break occurs at year "K", *T(k)* reaches a maximum quite close to the year k = K. The test statistic ${T}_{0}$ has the following definition (Eq. 7):

7 $${T}_{0}=\mathrm{max}T\left(k\right), 1<k<n$$

When ${T}_{0}$ exceeds the dependent on sample size critical value, rejecting the null hypothesis^101^.

*Von Neumann’s Ratio test:* was developed by him in 1941^100^. For an annual series ${X}_{i}$ (i is the year) with mean "X," The ratio of the successive mean squares is what is referred to as "N" given as (Eq. 8):

8 $$N= \sum_{i=1}^{n-1}({X}_{i}-{X}_{i+1}{)}^{2}/\sum_{i=1}^{n}({X}_{i}-\overline{X }{)}^{2}$$

The value of N typically falls short of the predicted value when the series is interrupted. The values of N may exceed two if there is a temporary fluctuation in the mean. This test cannot pinpoint the precise location of the break year.

### Method for trend analysis

The non-parametric Mann-Kendal (MK)^51,102^ test was used to find trends in rainfall. The MK test is frequently used to determine trends (if analysis occurs in meteorological data series)^50,60,103–106^. MK test is less sensitive to outline and suitable for detecting rainfall trends and has been applied to explore seasonal and annual rainfall trends^107^. At a 95% confidence level, the trend test was performed for each station, and the Sens slope technique^108^ was used to evaluate the trend's magnitude^105^. Data were checked by the tests’ parameters before performing the MK test to find precipitation patterns across the time series from chosen stations.

#### M–K test

Kendall^109^ established the MK test, first developed by Mann^110^, as a nonparametric test for trend identification and a test statistic. A two-tailed MK test is frequently used to determine if an outcome value tends to rise or fall over time.

The MK test treats ${y}_{i}$ and ${y}_{j}$ as two subsets of the time series of the n data, where i and j refer to years. j refers to one added year with i.

As a result, the MK Statistic (S) specified in Eqs. (9), (10), and (11) are as follows^58,102^.

9 $$\mathrm{S}=\sum_{j=1}^{n-1}\sum_{j=i+1}^{n}\mathrm{sign}({y}_{j}-{y}_{i})$$

where,

10 $$\mathrm{Sign }\left({y}_{j}-{y}_{i}\right)=\left\{\begin{array}{c}1\, if \,{y}_{j}-{y}_{i}>0\\ 0\, if \,{y}_{j}-{y}_{i}=0\\ -1 \,if\, {y}_{j}-{y}_{i}<0\end{array}\right\}$$

${\mathrm{y}}_{\mathrm{j}}$ is the yearly value for j year, and ${\mathrm{y}}_{\mathrm{i}}$ is the yearly value for i year (Eq. 10).

11 $$\mathrm{Var }\left(\mathrm{S}\right)=\frac{\left[\mathrm{n}\left(\mathrm{n}-1\right)\left(2\mathrm{n}+5\right)- \sum_{\mathrm{t}}\mathrm{t}\left(\mathrm{t}-1\right)\left(2\mathrm{t}+5\right)\right]}{18}$$

In this case, ‘t’ represents the range of any hypothetical tie of sample points. The $\sum t$ indicates the total of all ties. As a result, the sample size is more than ten, and Eq. 12 estimates the regular standard input 'Z' value.

12 $$\mathrm{Z}=\left\{\begin{array}{c}\frac{S-1}{\sqrt{Var(S)}} \,if \,S>0\\ 0 \, if \,S=0\\ \frac{S+1}{\sqrt{Var(S)}} \, if \, S<0\end{array}\right\}$$

The upward (growing) trend is therefore shown by positive values of “Z,” whereas the downward (decreasing) trend is shown by negative values of “Z”^111^. The significance threshold for the current study was α = 0.05, which had a 95% confidence level.

#### Sen’s slope estimator

Sen's slope estimator begins by calculating the slope ($${T}_{i})$$ of each data pair using Eq. 13^108^;

13 $${\mathrm{T}}_{\mathrm{i}}=\frac{{\mathrm{X}}_{\mathrm{j}}-{\mathrm{X}}_{\mathrm{k}}}{\mathrm{j}-\mathrm{k}}$$

here, i = year = 1, 2, 3, …n.Where ${X}_{j}$ and ${X}_{k}$ stand for the variables' respective times (j > k). In the examination of historical time series, ${T}_{i}$>0 denotes an upward and ${T}_{i}$<0 denotes a downward trend.

#### Method for change point detection

Any trend detection research would be lacking without mentioning when the trend changed^97^. This stud used the Pettitt test^112^ and Cumulative Sum test to investigate if any weather stations of annual rainfall include any abruptly changing transition points.

*Pettit test:* The resulting test statistics are stated in Eq. 14 when the duration of the study period is denoted by *t* and the shift occurs at *m* years^113^.

14 $${U}_{t, m}=\sum_{i=1}^{m}\sum_{t+1}^{t}sgn({k}_{i}-{k}_{j})$$

Here, $sgn({k}_{i}-{k}_{j})$ is calculated from Eq. 15

15 $$sgn\left({k}_{i}-{k}_{j}\right)=\left\{\begin{array}{ccc}1& if \, ({K}_{i}-{K}_{j})& >1\\ 0& if\, ({K}_{i}-{K}_{j}) & =0\\ -1& if \, ({K}_{i}-{K}_{j})& <1\end{array}\right\}$$

All randomly generated variables from 1 to m are used to determine ${U}_{t, m}$. Where the test statistic's magnitude is located ${U}_{t, m}$ is largest, the bulk of unique change points are identified (Eq. 16). When ${U}_{t, m}$ is at its highest, the likelihood of a shifting year is assessed (Eq. 17).

16 $${Z}_{T}=Max{U}_{t, m} 1\le t\le m$$

17 $$P=1-exp\left(\frac{-6{Z}_{T}^{2}}{{K}^{2}+{K}^{3}}\right)$$

The null hypothesis is considered to be rejected if the *p*-value falls below the significance level.

*Cumulative Sum (CUSUM) test:* The distribution-free CUSUM test^114^ determines if variations in the means between two portions of a series are different for an indefinite period. The maximal CUSUM value of the k serves as the test statistic, ${V}_{k}$. Equation 18 is used for calculating ${V}_{k}.$

18 $${V}_{k}= \sum_{i=1}^{k}sign({x}_{i}-{x}_{median})$$

where, k = 1, 2, 3, ….. , n, and $sign({x}_{i}-{x}_{median})$ is given as Eq. 19.

19 $$sign\left({x}_{i}-{x}_{median}\right)= \left\{\begin{array}{ccc}-1& if ({x}_{i}-{x}_{median})& <0\\ 0& if ({x}_{i}-{x}_{median})& =0\\ 1& if ({x}_{i}-{x}_{median})& >0\end{array}\right\}$$

### Innovative trend analysis (IAA) methods

The most significant benefit of the IAA over the MK test is that it not requires any presumptions such as non-linearity, serial correlation, and sample sizes^115^. This widely used IAA^115,116^ was proposed by Şen^117^. As for IAA the rainfall data classified into two periods: these are (a) 1981–2000, (b) 2001–2020. First period represents as ${X}_{i}$ (X-axis), and the second period ${Y}_{i}$ (Y-axis). The data shows a stable trend when it is displayed along the 1:1 line. A rising trend was observed when data was plotted above the 1:1 line, and a declining trend was observed when data was plotted under the 1:1 line^117,118^.

The equation behind IAA^119^ are (Eq. 20):

20 $$s= \frac{1}{n}\sum_{i=1}^{n}\frac{10(\overline{y }-\overline{x })}{n}$$

where, $\overline{x }$ and $\overline{y }$ = arithmetic average of ${X}_{i}$ and ${Y}_{i}$ series. n = number of observations. A rising trend is represented by a positive s value, and a descending trend is represented by a negative s value. After that, the indication is multiplied by 10 to compare with the MK test^120^.

### Method for analyzing rainfall changes

The impact of changes in atmospheric circulation on rainfall trend patterns has been measured. Using the Pettit test for the period 1981–2020 in Bangladesh, we first discovered a big shift in the yearly mean rainfall that occurred recently and noted that the change point occurred after 2000. Using the European Centre for Medium Range Weather Forecasts (ECMWF) ERA5 reanalysis data, the two periods before and after the modifications’ respective changes in circulation are then calculated by deducting 1981–2000 from 2001 to 2020.

### Method for rainfall prediction

*Multilayer Perception (MLP):* is an inverse traditional prediction method in meteorology based on self-adaptive mechanisms^75^ comprised of input, output, and hidden layers. These three layers (Fig. 4) are used for short-term, mid-term, and long-term predicting of meteorological research^121^. The MLP is the most often used ANN design in hydrologic modelling^139^, used for short-term rainfall prediction^143–145^, also more than 15 years^27^.

#### Input attributes in the prediction model

Previous studies for rainfall prediction using multilayer perceptions used temperature, humidity, solar radiation, and wind speed as rainfall predictors^143–145^. The input attributes as the rainfall predictors are shown in Table 1.

**Table 1:** Rainfall parameters used in our study and their corresponding measurement units.

Rainfall predictors	Unit	Max	Min	Mean
Minimum temperature	°C	36.43	19.6	30.12
Maximum temperature	°C	27.33	8.19	20.08
Relative humidity	% (percentage)	83.35	55.81	71.68
Solar radiation	W/m^2^	273.56	75.81	168.32
Wind speed	m/s	3.54	0.18	0.94

We split the entire rainfall dataset into training and testing datasets for the current investigation. After normalizing the data, we used the Multi-layer Perceptron neural network to predict the monthly rainfall using the R software version 4.2.2 (https://www.r-project.org/). We have compiled the rainfall data for Bangladesh from 28 stations. The next step after collecting the normalized data is to train the model using the input data in Matlab version 9.9 (https://www.mathworks.com/products/matlab.html). Only 70% of the input data are used by the algorithm for training. Only 336 of the 480 samples are used for training, and these are chosen at random from the data set. The average monthly rainfall data from 1981 to 2020 are used as an input in this prerdiction method. The output layer consists the predict monthly rainfall. We validate the predicted monthly rainfall of 24 month (2021–2022) with the actual rainfall.

#### Evaluating the skill score of MLP

**Figure 2:**
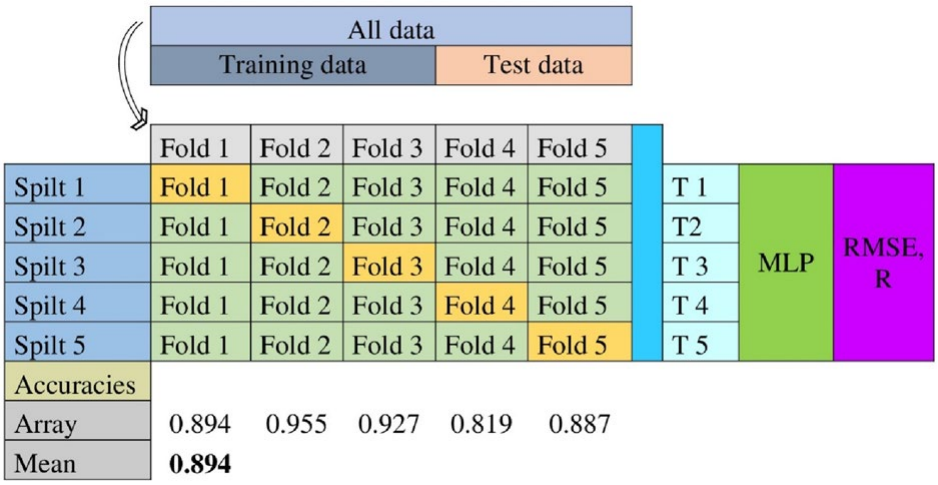
The function of K-fold cross-validation technique and accuracy level of MLP.

We have evaluated the skill score of MLP using K-fold cross validation technique in Python version 3.10.12 (https://www.python.org/). The K-fold cross-validation approach is highly beneficial for analyzing the attributes and variance of the input dataset for an MLP model^137^. The K-fold cross validation model run over the monthly rainfall of Bangladesh from 1981 to 2020. The cross-validation function and multiple metric evaluation are shown in Fig. 2. Two arrays make up each fold: one is connected to the training set, and the other is related to the test set. Testing and validation consume 30% (144 samples) of the input data.

To find the optimum MLP model for predicting rainfall, we repeatedly applied the model to the same precipitation datasets. Five evaluations of the model are performed, with each evaluation utilizing a different fold as the validation set. The accuracies for all data from the five arrays are observed 0.894, 0.955, 0.927, 0.819, and 0.887 respectively against the 1.00. The mean accuracy is 0.894, it means 89.4%. The skill score of this MLP model from K-fold cross-validation is more than 89%. Since the MLP model performed well between 1981 and 2020, we may extend its results to the year 2030.

Additionally, we used Root Mean Square Error (RMSE) and correlation methods to assess how well MLP performed in terms of prediction.

#### Step in 95% range of prediction construction using bootstrap MLPs

**Figure 3:**
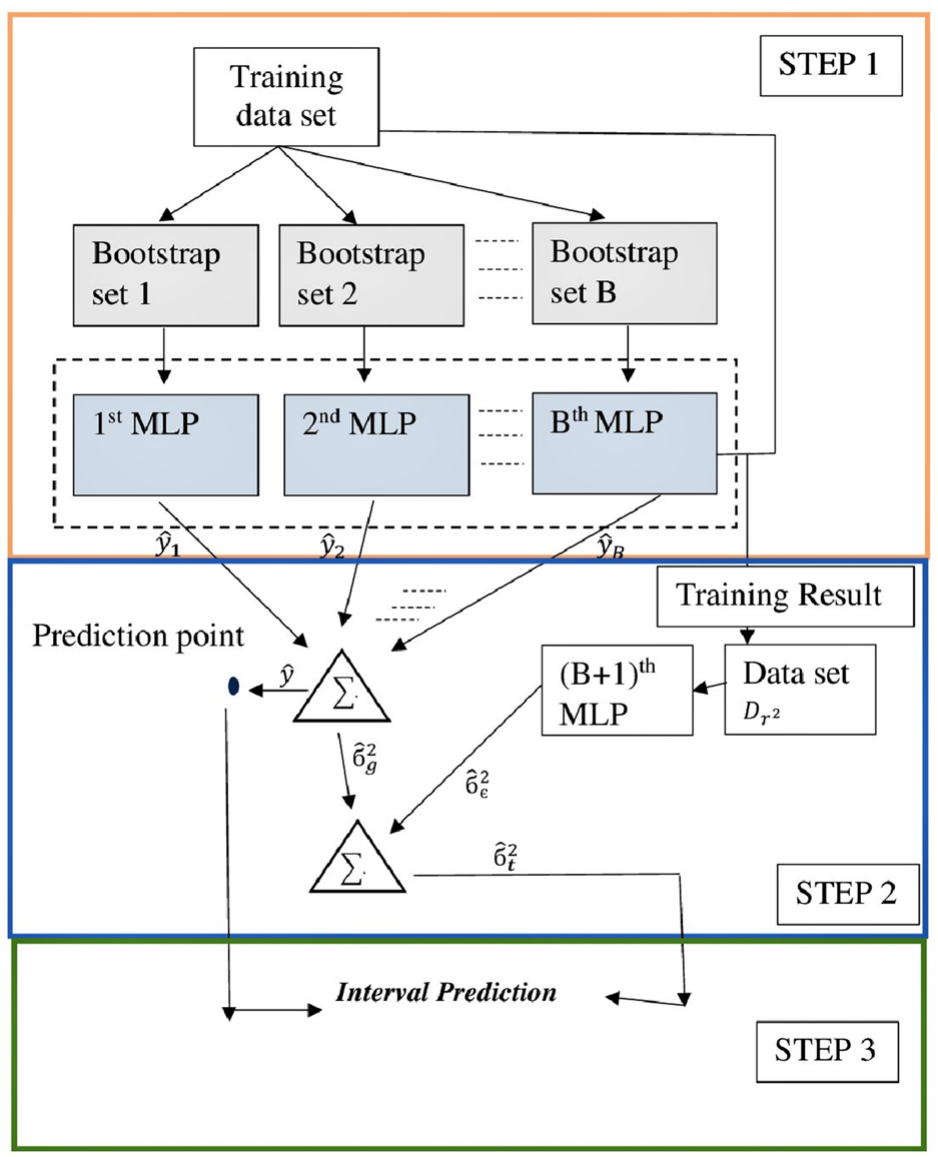
The framework of bootstrap-MLPs based PI constructions.

The paired bootstrap approach^140^ was used to calculate model uncertainty in regression models. This study carries an ensemble of more than 1000 model runs using the bootstrapping technique to get the 95% range of the predictions. As shown in Fig. 3, the following is a description of how Prediction Intervals (PI) based on the bootstrap-MLPs approach is constructed:

**Step1**: Split the original data set $D= {\left\{\left({X}_{i}, {P}_{i}\right)\right\}}_{i=1}^{N}$ into training ${D}_{train} = {\left\{\left({X}_{i}, {P}_{i}\right)\right\}}_{i=1}^{{N}_{train}}$ and testing ${D}_{test} = {\left\{\left({X}_{i}, {P}_{i}\right)\right\}}_{i=1}^{{N}_{test}}$. Generate B training data sets which are each constructed using pairs bootstrapping method based on the initial training data set ${D}_{train} = {\left\{\left({X}_{i}, {P}_{i}\right)\right\}}_{i=1}^{{N}_{train}}$. Train B MLPs using B bootstrapped training data sets.

**Step2**: Estimate the true regression $\widehat{y}({X}_{i})$, model uncertainty  , and noise  .

(1) The estimate of true regression $\widehat{y}({X}_{i})$ can be calculated as follows (Eq. 21):

21 $$\widehat{y}\left({X}_{i}\right)=\frac{1}{B}\sum_{b=1}^{B}{\widehat{y}}_{b}({X}_{i})$$

where, ${\widehat{y}}_{b}({X}_{i})$ is the prediction value generated by the *b* thMLP.

(2) The estimate of the variance in the MLP model uncertainty  can be obtained from (Eq. 22):22

(3) According to  , the variance of the noise  can be estimated as follows^141^ (Eq. 23):23

A set of variances squared residuals ${r}^{2}{(X}_{i})$ are calculated to estimate a model to fit the remaining residuals (Eq. 24):24

Afterward, a new data set ${D}_{{r}^{2}}$ can be built as follows (Eq. 25):

25 $${D}_{{r}^{2}}=({X}_{i, {r}^{2}}({X}_{i})), i =1, ..... , N$$

Based on ${D}_{{r}^{2}}$, a separate MLP model can be trained to estimate the variance of the noise  . An exponential activation function should be adopted in the new neural network model to ensure a positive variance^142^.

**Step 3** Construct PIs with (1 − a) × 100% Prediction Interval Nominal Confidence (PINC) using the upper and lower boundary of the PI.

In this study, we calculate 80% and 95% intervals. The predicted errors have a normal distribution. A total of 95% and 80% prediction intervals for the h -step prediction is respectively in Eqs. 26 and 27.2627here,  is an estimate of the standard deviation of the h -step prediction distribution.

#### Multi-layer perceptron strategy

**Figure 4:**
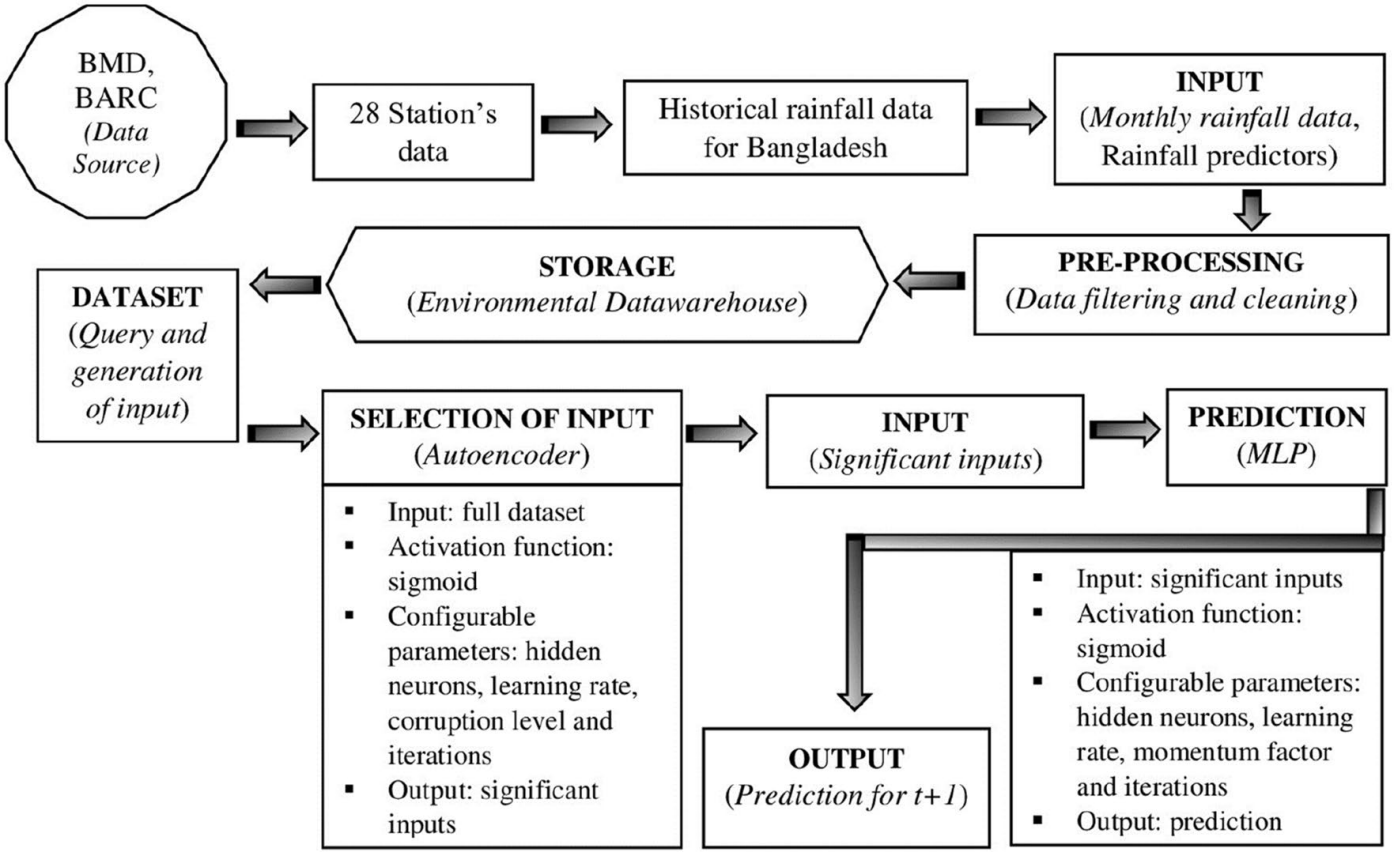
Flow of proposed prediction methodology.

This study used the MLP deep learning approach to predict rainfall. To predict time series, auto-encoders conduct feature extraction in MLP^69^. First, all non-linear characteristics are extracted from the data using auto-encoders. Figure 4 depicts the suggested methodology's flow.

**Figure 5:**
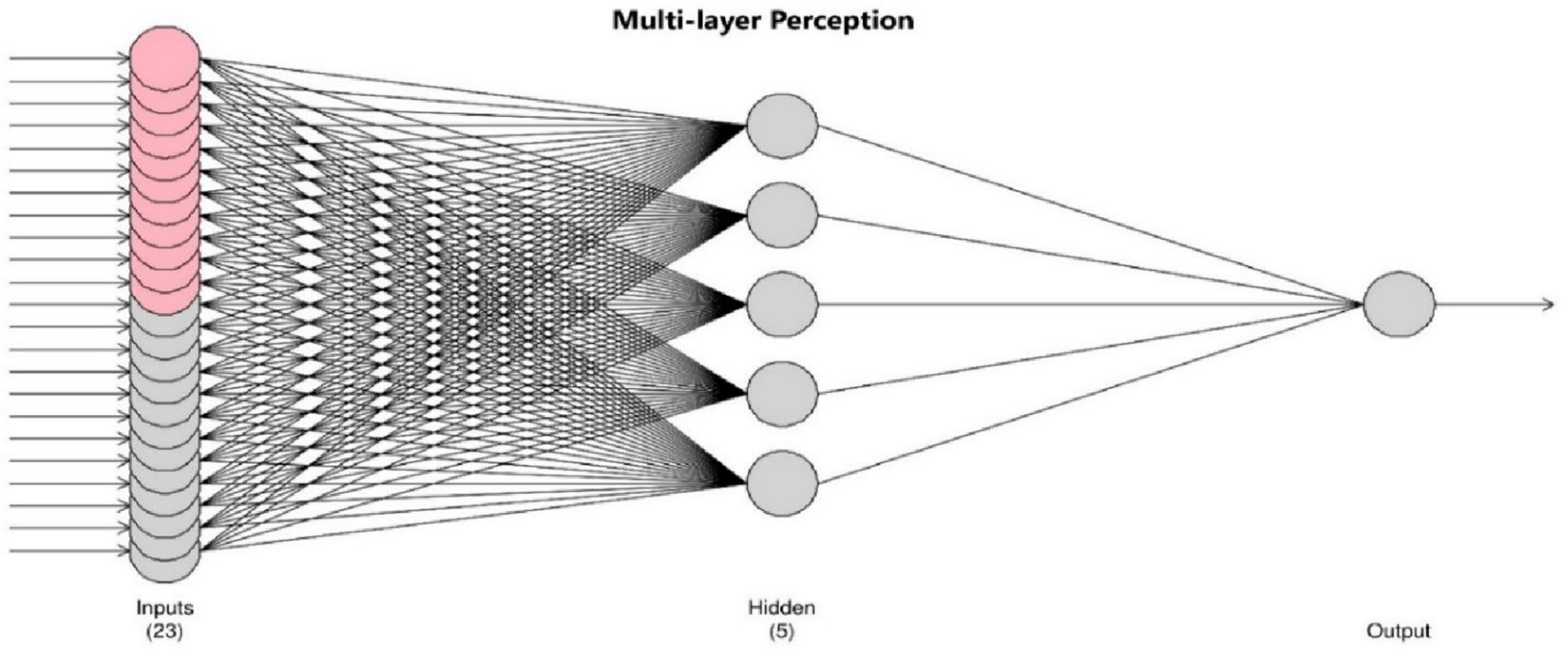
Input, hidden, and output layers in multilayer perceptron neural network.

This auto -encoder consists of three layers: Input Layer, Hidden Layer, and Output Layer. Hidden layers performing intermediary computations are shown in Multi-layer Perceptron (MLP) Fig. 5.

*Input layer:* There are 23 neurons (${X}_{i}, i=1, 2, 3,\dots .,23.)$ in the input layer. In the input layer, the neurons are arranged in the manner shown in Eq. 28.

28 $$ a_{(j - 10)}^{1} 1,\,a_{(j - 9)}^{1} 1,\,a_{(j - 8)}^{1} 1,\,a_{(j - 7)}^{1} 1,\,a_{(j - 6)}^{1} 1,\,a_{(j - 5)}^{1} 1,\,a_{(j - 4)}^{1} 1,\,a_{(j - 3)}^{1} 1,\,a_{(j - 2)}^{1} 1,\,a_{(j - 1)}^{1} 1,\,a_{j}^{1} 1,a_{(j - 11)}^{1} 2,\,a_{(j - 10)}^{1} 2,\,a_{(j - 9)}^{1} 2,\,a_{(j - 8)}^{1} 2,\,a_{(j - 7)}^{1} 2,\,a_{(j - 6)}^{1} 2,\,a_{(j - 5)}^{1} 2,\,a_{(j - 4)}^{1} 2,\,a_{(j - 3)}^{1} 2,\,a_{(j - 2)}^{1} 2,\,a_{(j - 1)}^{1} 2,\,a_{j}^{1} 2 $$

*Hidden layer:* There are 5 hidden layers altogether. Every neuron is related to every hidden layer. The hidden layer was calculated using (Eq. 29).

29 $${Y}_{k}= \int {\sum }_{i}{X}_{i}\times {W}_{jk}+{B}_{k}$$

Here, ${W}_{jk}$ is the weight connecting the *i* th input layer neuron to the *k* th neuron of hidden layer. ${B}_{k}$ is the bias adden to *k* th neuron of hidden layer.

*Output layer:* In the output layer, there is just one neuron, and its value is calculated using (Eq. 30):

30 $$Z= \int {\sum }_{m}{Y}_{m}\times {W}_{m}+B$$

#### Accuracy measurement

**RMSE:** The optimal model has a minimal root mean square error (RMSE), which calculates the standard deviation of the random components in the data (Eq. 31).

31 $$\mathrm{RMSE }= \sqrt{\sum_{t=1}^{n}\frac{{\left[{y}_{t}\left(obs\right)-{y}_{t}(pred)\right]}^{2}}{n}}$$

*PBIAS*: The percent of bias is the average tendency for the predicted output to differ from the observed data either more or less. Low PBIAS values (Eq. 32) imply accurate model simulations; the ideal value is 0.0. The index of agreement, which ranges from 0 (no correlation) to 1, assesses the level of model prediction inaccuracy (perfect fit).

32 $$\mathrm{PBIAS }(\mathrm{\%}) =\frac{\sum_{\mathrm{i}=1}^{\mathrm{N}}({\mathrm{O}}_{\mathrm{i}}-{\mathrm{P}}_{\mathrm{i}})-100}{\sum_{\mathrm{i}=1}^{\mathrm{N}}{\mathrm{O}}_{\mathrm{i}}}$$

${O}_{i}$, and ${P}_{i}$ stand for the observed data, simulated data from the model, and observed mean, respectively.

**Correlation**: are calculated as^144^ (Eq. 33).

33 $$R = \frac{\sum_{i=1}^{N}({y}_{t}^{o}-\overline{{y }^{o}})({y}_{t}^{c}-\overline{{y }^{c}})}{\sqrt{\sum_{t=1}^{N}\left[{(y}_{t}^{o}-\overline{{y }^{o}}{)}^{2}\right]\left[{(y}_{t}^{c}-\overline{{y }^{c}}{)}^{2}\right]}}$$

where ${y}_{t}^{o}$ and ${y}_{t}^{c}$ represent the observed and calculated values at time t respectively, $\overline{{y }^{o}}$ and $\overline{{y }^{c}}$ yc represents the mean of the observed and calculated values.

### Calculation method for change rates in data from a time series of rainfall

The straightforward statistical approach of percentage change is used to identify the yearly and seasonal rainfall change rates for the pre-change and post-change sites. This technique is pretty straightforward, yet it does its job quite well. Equation 34 is used to compute it.

34 $$Change\left(\%\right)over period= \left(\frac{Average\, rainfall \,for \,post\, change \,point-Average \,rainfall \,for\, pre \, change \,point}{Average\, rainfall\, of\, pre\, change \,point}\right)\times 100$$

### Methods for rainfall variations

Post-monsoon and monsoon precipitation and moisture divergence for the period 1980–2020 were obtained on 1.25° × 1.25° grids from the European Centre for Medium-Range Weather Forecasts (ECMWF), ERA-5 (http://apps.ecmwf.int/ datasets/data/interim-full-daily). The most current ocean-atmospheric changes reanalysis datasets available from 1980 onwards are in the ERA5-Interim. To evaluate the impact of cloud cover on rainfall variation, a low cloud cover dataset was also constructed from the ECMWF ERA5 data^44^. The impact of changes in atmospheric circulation on rainfall trend patterns has been measured. First, using the Pettit test for the years 1980 to 2020 in Bangladesh, we found a recent significant change point in the annual mean rainfall and noted that the change point occurred after 2001. Then, using the ECMWF ERA5 reanalysis data, the difference in circulation between the two eras before and after the adjustments is quantified by subtracting 1980–2001 from 2002 to 2020. The spatial maps were created using the GrAd software.

### Spatial mapping method

In this work, the Inverse Distance Weighting method was used to interpolate the data to create a spatial rainfall map.

*IDW:* The IDW interpolation is based on Eq. 35^122^.

35 $$F \left(x, y\right)=\sum_{i-2}^{n}{w}_{i}{f}_{i}$$

When each scatter point is given a weight function ${w}_{i}$. and ${f}_{i}$ are the specified function values at the scatter points. The collection's overall total of scatter points is n.

The weight function's traditional configuration is calculated from Eq. 36 similar as Monir et al.^102^.

36 $${{\varvec{W}}}_{{\varvec{i}}}=\frac{{{\varvec{h}}}_{{\varvec{i}}}^{-{\varvec{p}}}}{\sum_{{\varvec{j}}=1}^{{\varvec{n}}}{{\varvec{h}}}_{{\varvec{j}}}^{-{\varvec{p}}}}$$

Here ${h}_{i}$ is the distance between the scatter and interpolation points. The power parameter, p, is a freely selected positive real value (Eq. 37).

37 $$\text{Interpolation point }{h}_{i}=\sqrt{{(x-{x}_{i})}^{2}+ {(y-{y}_{i})}^{2}}$$

where (x, y) stands for the interpolation point's coordinates. and ($${x}_{i},{y}_{i})$$ for the coordinates of the scatter point’s coordinate.

## Results

### Rainfall pattern in Bangladesh

The average annual rainfall in Bangladesh was observed at 2432.6 mm in this study period. The highest average annual rainfall was observed in Teknaf (4212.9 mm) and the lowest in Ishurdi (1466.4 mm). On average, 442.8 mm of rainfall occurs in the pre-monsoon, 1905.3 mm during the monsoon, and 84.5 mm in the post-monsoon.

**Figure 6:**
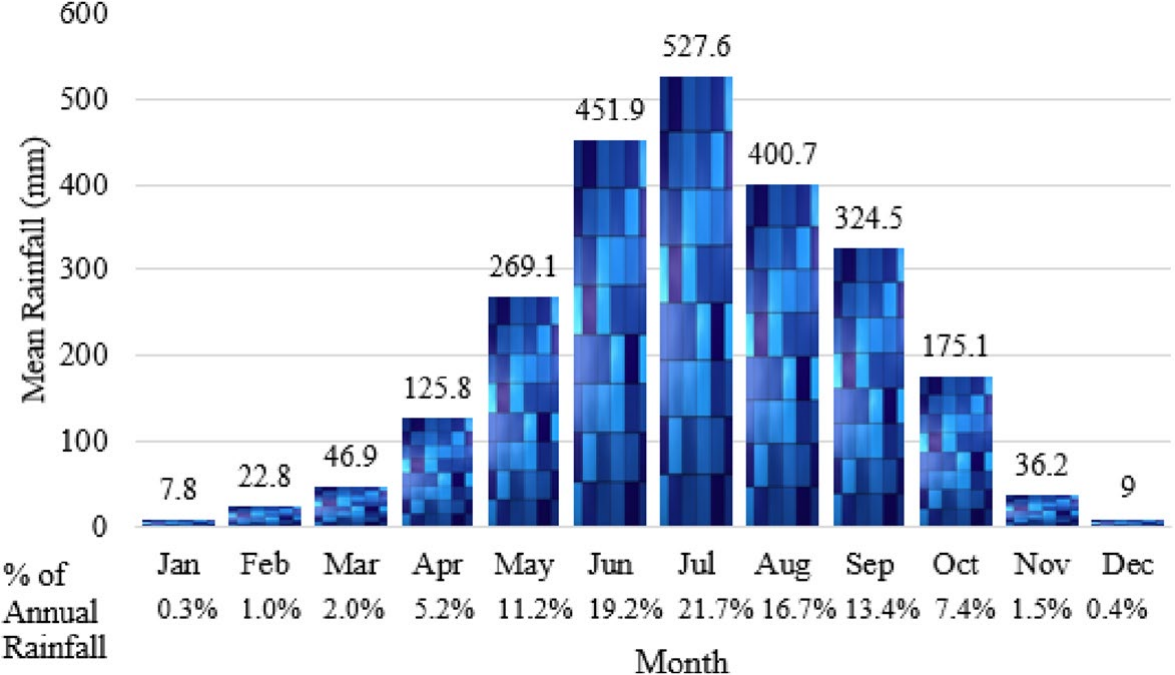
Monthly mean rainfall (1981–2022) in Bangladesh.

In Bangladesh, according to data on average rainfall from 1981 to 2020, June to September had the most rainfall (Fig. 6). July recorded the highest average rainfall (520.59 mm). November through February have the least rain (Fig. 6). June, July, August, and September were observed, respectively, 19.2%, 21.7%, 16.7%, and 13.5% of annual rainfall. On the other hand, 1.5% rainfall was recorded in November, 0.4% in December, 0.3% in January, and 1.0% in February (Fig. 6).

Results on average seasonal rainfall (1981–2020) in Bangladesh are presented in Table 2. Among the 28 monitoring stations, the average pre-monsoon season rainfall is from 279 mm (Satkhira, which is situated in Bangladesh's southwest) to 1076.1 mm (The northeastern region's Sylhet station) (Fig. 1). The most rainfall during the monsoon season (3754.5 mm) was recorded in Teknaf. The lowest rainfall was recorded in Ishurdi (1133.1 mm). During the post-monsoon, the highest rainfall (120.9 mm) was in Cox's Bazar, and the lowest (40.8 mm) was in Rangpur. Compared to earlier seasons, where the monsoon faced excellent rainfall, the post-monsoon season had relatively little rainfall (Table 2).

**Table 2:** Average seasonal rainfall (1981–2020) in Bangladesh.

Weather monitoring station	Average seasonal rainfall (mm)			Weather monitoring station	Average seasonal rainfall (mm)		
	Pre-monsoon	Monsoon	Post-monsoon		Pre-monsoon	Monsoon	Post-monsoon
Dhaka	456	1482.9	67.5	Barisal	366	1597.5	96.9
Mymensingh	486.9	1669.8	50.7	Bhola	386.4	1722.6	92.4
Faridpur	360.3	1262.1	74.4	Khepupara	373.5	2326.8	113.1
Madaripur	383.7	1486.2	71.4	Patuakhali	378	2089.2	100.2
Srimangal	716.4	1527.9	87.9	Chandpur	480.6	1588.2	87.3
Sylhet	1076.1	2850	86.7	Teknaf	350.4	3754.5	108
Bogra	300	1365.3	44.1	Chittagong	483.3	2290.8	96.9
Rajshahi	242.7	1196.4	44.4	Comilla	518.4	1460.4	89.1
Ishurdi	277.2	1133.1	56.1	Cox's Bazar	453.9	3023.7	120.9
Dinajpur	294.9	1497	44.1	Feni	555.9	2318.4	98.7
Rangpur	419.1	1761.6	40.8	M.Court	539.4	2428.8	102.9
Jessore	294.9	1270.5	84.6	Rangamati	511.5	1933.8	104.4
Khulna	303.6	1417.2	99.6	Sandwip	552.9	2964.9	105.3
Satkhira	279	1327.8	95.1	Sitakunda	558	2601.9	101.4

*Source: BMD, 2021.*

**Figure 7:**
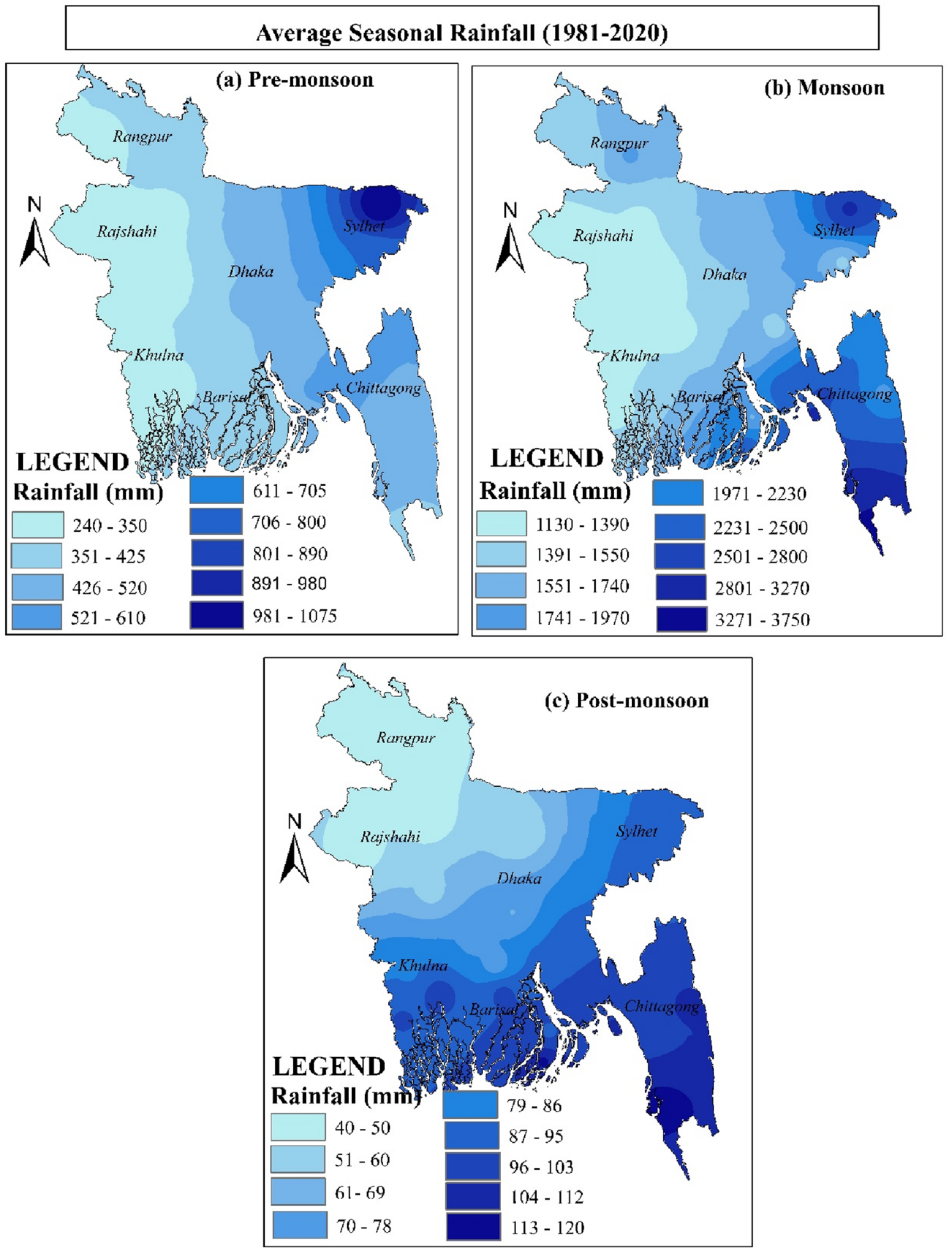
Spatial distribution of average seasonal rainfall (1981–2020) in Bangladesh: (**a**) pre-monsoon rainfall, (**b**) monsoon rainfall, and (**c**) post-monsoon rainfall, prepared by the authors using ArcGIS software version 10.5, (https://www.esri.com/en-us/arcgis/products).

Figure 7 shows the spatial distribution of average seasonal rainfall (1981–2020) over Bangladesh. Pre-monsoon rainfall varies, being lowest in the west and most significant in the northeast (Fig. 7a). The northeast and southern regions had the maximum rainfall during the monsoon season (Fig. 7b).

In contrast, the northern region has the least rainfall during the post-monsoon season (Fig. 7c). The seasonal changes affect the geographic variation of Bangladesh's average rainfall (Fig. 7).

### Monthly rainfall trends

The *MK Z* values for monthly rainfall (1981–2020) over Bangladesh with a 95% confidence level are presented in Table 3. The negative values represent a decline in rainfall, where *Z* < − 1 represents the significant negative trend. Similarly, the positive values represent a rising trend in rainfall, and when the value of *Z* is more than 1, this is a significant rising trend.

**Table 3:** The trend in monthly rainfall over Bangladesh from 1981 to 2020 (*P* > 0.05).

Weather monitoring station	*MK Z*											
	Jan	Feb	Mar	Apr	May	Jun	Jul	Aug	Sep	Oct	Nov	Dec
Dhaka	− 1.76	− 1.1	− 1.59	− 1.04	− 1.11	− .08	.99	.4	− 2.84	− .57	− 1.06	.97
Mymensingh	− .83	− .75	− .61	.02	− .82	.48	− 1.2	− .73	− 1.71	− 1.36	.5	− 1.77
Faridpur	1.14	− 1.22	− 2.38	− 1.89	− 1.94	.25	1.49	.15	− .82	.34	− .22	− .05
Madaripur	− .96	− .89	− 1.27	− 2	− 1.52	− 1.79	− .47	− 1.33	− .5	.54	− .11	.2
Srimangal	.05	− 1.06	− .94	.04	1.4	1.13	.23	− .62	− 1.75	0	− 1.32	− .59
Sylhet	− 1.28	− .62	− 1.68	− .27	1.01	.05	− 2.11	− .33	− 2.18	− .3	− .48	− 1.6
Bogra	− .43	− .71	.15	.55	− 1.1	− .92	− 1.74	− 1.08	− 2.52	− .08	− 1.62	− 2.08
Rajshahi	− .67	− 1.53	.29	− .09	1.85	1.04	− 0.01	− .59	0	− .61	− 3.07	− 1.65
Ishurdi	− .43	− 1.45	− .2	− .3	− 1.53	− .36	− .25	− .76	− 1.79	.49	− .6	− .81
Dinajpur	− .104	− .06	1.87	1.45	− .8	− .98	− 2.56	− 2.72	− 3.41	− .4	− .14	− 1.75
Rangpur	− .9	− .8	− .07	− .83	.97	− 1.27	− 1.31	− 1.1	− 2.16	− .41	− 1.94	− 2.69
Jessore	− 1.3	− .77	− .16	.02	− .8	− 1.31	2.03	− 1.21	− .73	− .2	0	− .49
Khulna	− 1.52	− .6	− .74	− 1.17	− .75	− .37	.98	− .21	.57	.2	− .67	− .97
Satkhira	− 1.38	− 1.12	− .16	− 2.28	.52	.13	.22	− 1.85	− .64	.48	− .77	− .23
Barisal	− 2.33	− 1.4	− 1.65	− .62	− .86	− .61	.3	− 1.74	.02	.25	− .56	− .26
Bhola	− 1.28	− 1.49	− .77	− 1.79	− .86	− 1.83	− .33	− .62	− .28	1.29	− .7	− .27
Khepupara	− .95	− .7	− .5	− 1.69	− .26	− .85	1.5	− .61	.28	1.76	− .32	.18
Patuakhali	− 1.1	− 1.31	− .43	− 1.76	− .83	− 1.71	.19	− 1.27	− .86	1.15	− .53	− 1.43
Chandpur	− 1.06	.33	− 1.14	− 1.01	− .32	− .01	− .23	− 1.19	− 1.26	.33	− .53	− 1.14
Teknaf	0	− 2.11	− .31	− .82	.66	− 1.41	− 1.4	1.13	− 1.35	1.08	− 2.89	− .16
Chittagong	− .36	− 1.59	− 1.64	− 2.09	.29	− .12	− .36	− .18	.65	.82	− 1.96	− 1.09
Comilla	− .97	− .73	− .85	− .27	− .45	.72	.45	− .19	− .51	− .48	− 1.09	.01
Cox's Bazar	− .7	− 2.14	− .06	− 1.89	.49	− .84	.54	− 1.07	.09	1.11	− 2.98	− .75
Feni	− 1.18	− 1.71	.16	− 1.56	− 1.49	− .05	− 1.32	− .51	− .12	1.32	− 1.23	− 1.35
M.Court	− .7	− .61	− .99	− 1.13	− .21	− .36	− .82	− 2.83	− .4	1.7	− .42	− .75
Rangamati	− .23	− 1.63	− .71	− .65	− .13	1.59	.18	− .93	.85	1.84	− .79	− .69
Sandwip	− .43	− 1.01	− .76	− 1.89	.75	.47	1.48	.83	− .29	.69	− 1.49	− 1.1
Sitakunda	.51	− 1.75	− 1.98	− .75	− .42	.92	.75	.34	.15	− .25	− 2.08	− .07

**Figure 8:**
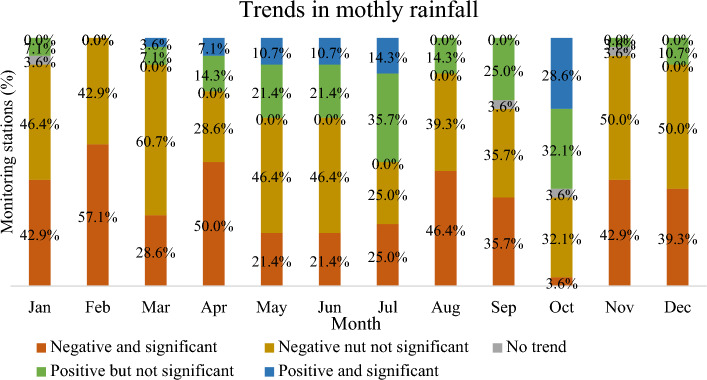
Trend of the monthly rainfall represented as a percentage of the rainfall monitoring stations.

According to the MK Z value, a significant amount of monitoring stations has a declining trend in rainfall for all of the months. Figure 8 shows that February observed a declining trend for all monitoring stations. More than 80% of monitoring stations face a declining trend for January, March, August, November, and December. April, May, June, and September observed 60–80% monitoring stations for a declining trend. Also, more than 50% of monitoring stations face a significant declining trend for February and April. On the other hand, only 2 months (July and October) have observed a rising trend for more than 50% of monitoring stations. A significant rising trend (28.6%) was found only for October (Fig. 8).

### Trends in seasonal rainfall

Over the years 1981 to 2020, the seasonal rainfall MK trend value and projected Sen's slope value are shown in Table 4. All of the monitoring stations have a declining trend for the post-monsoon season. During the pre-monsoon and monsoon, roughly 82% and 75% of the monitoring stations exhibit a declining tendency in rainfall (Table 4).

**Table 4:** MK trend and estimated Sen’s slope for seasonal rainfall from 1981 to 2020.

Weather monitoring station	MK (*Z)* value			Sen’s slope value		
	Pre-monsoon	Monsoon	Post-monsoon	Pre-monsoon	Monsoon	Post-monsoon
Dhaka	− 1.97	− 1.26	− 1.91	− 0.18	− 0.17	− 0.05
Mymensingh	− 0.73	− 2.09	− 1.10	− 0.08	− 0.40	− 0.02
Faridpur	− 2.65	− 0.38	− 1.56	− 0.21	− 0.07	− 0.04
Madaripur	− 1.83	− 0.78	− 0.94	− 0.14	− 0.19	− 0.02
Srimangal	0.80	− 0.22	− 1.01	0.09	− 0.04	− 0.03
Sylhet	1.25	− 2.09	− 1.39	0.05	− 0.39	− 0.35
Bogra	− 1.13	− 1.67	− 1.32	− 0.09	− 0.38	− 0.02
Rajshahi	1.48	− 0.22	− 2.58	0.07	− 0.03	− 0.03
Ishurdi	− 0.87	− 0.87	− 1.43	− 0.05	− 0.13	− 0.03
Dinajpur	− 0.36	− 3.25	− 1.19	− 0.02	− 0.77	− 0.01
Rangpur	1.22	− 2.58	− 2.87	0.10	− 0.56	− 0.02
Jessore	− 0.80	− 0.48	− 1.65	− 0.04	− 0.09	− 0.04
Khulna	− 0.87	0.90	− 1.18	− 0.04	− 0.09	− 0.04
Satkhira	− 0.66	− 0.76	− 0.77	− 0.03	− 0.09	− 0.03
Barisal	− 0.79	− 0.58	− 1.45	− 0.05	− 0.09	− 0.03
Bhola	− 1.34	− 0.92	− 1.29	− 0.12	− 0.14	− 0.04
Khepupara	− 1.15	2.37	− 0.18	− 0.08	0.34	− 0.01
Patuakhali	− 1.46	− 1.07	− 1.41	− 0.09	− 0.18	− 0.04
Chandpur	− 0.97	− 0.48	− 0.71	− 0.10	− 0.08	0.02
Teknaf	0.52	0.97	− 3.08	0.06	0.01	0.10
Chittagong	− 1.76	0.06	− 2.55	− 0.16	0.01	− 0.08
Comilla	− 1.06	− 0.26	− 0.72	− 0.09	− 0.05	− 0.02
Cox's Bazar	0.12	− 0.47	− 3.12	0.01	− 0.15	− 0.11
Feni	− 2.33	− 0.72	− 1.21	− 0.20	− 0.16	− 0.03
M.Court	− 1.34	− 1.37	− 1.17	− 0.01	− 0.32	− 0.04
Rangamati	− 0.87	1.41	− 2.06	− 0.11	0.27	− 0.07
Sandwip	− 0.20	1.83	− 1.94	− 0.03	0.54	− 0.06
Sitakunda	− 1.13	0.71	− 1.28	− 0.11	0.23	− 0.05

During the pre-monsoon, monsoon, and post-monsoon, approximately 43%, 29%, and 82% of monitoring stations have a significant declining trend (Z < − 1) (Table 4). However, just 8% of the monitoring stations that monitored the monsoon saw a discernible growing trend (Table 4).

**Figure 9:**
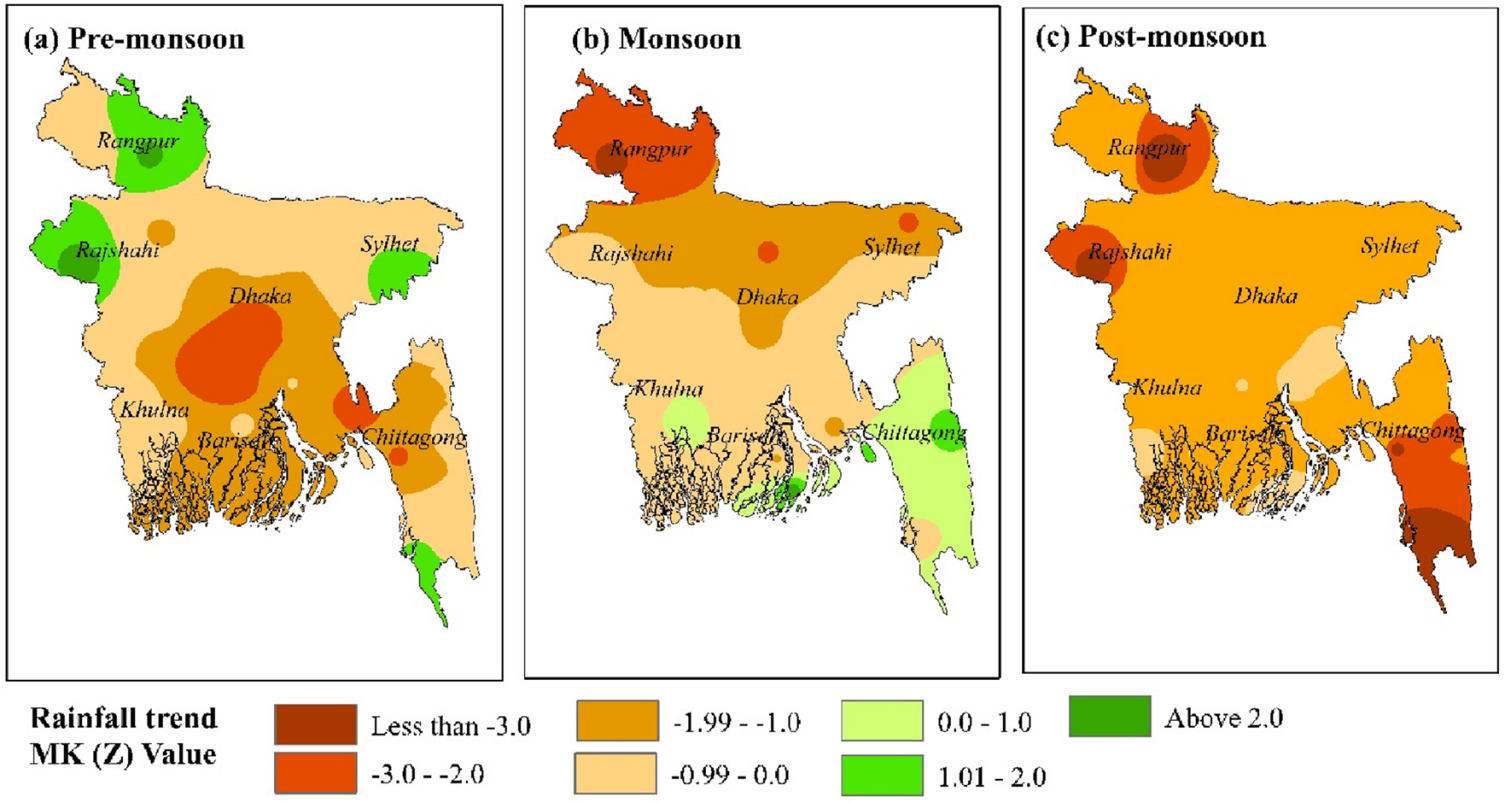
Spatial distribution of seasonal rainfall trends over Bangladesh from 1981 to 2020: (**a**) pre-monsoon rainfall trend, (**b**) monsoon rainfall trend, and (**c**) post-monsoon rainfall trend, prepared by the authors using ArcGIS software version 10.5, (https://www.esri.com/en-us/arcgis/products).

During the pre-monsoon, the central part of Bangladesh observed a rapid decline in rainfall. There is an upward tendency in the northern (Rangpur), western (Sylhet), eastern (Rajshahi), and far southern (Cox's Bazar) regions. The rest of the areas have a moderate decline in pre-monsoon rainfall (Fig. 9a). For the monsoon season, the rainfall trend varies a little rising in the southern part to declining in northern Bangladesh (Fig. 9b). All of Bangladesh observed a decline in post-monsoon rainfall (Fig. 9c). There is a significant decline rate in the northern (Rangpur), eastern (Rajshahi), and far southern (Cox's Bazar) regions. In contrast, this area significantly rose during the pre-monsoon season (Fig. 9a,c). This is a clear example of the rain delay.

#### Changepoint wise annual variation

On the datasets of seasonal rainfall's pre- and post-change points in each of the weather monitoring stations, we conducted the MK test. MK Z values for pre and post-change points are presented in Table 5.

**Table 5:** Trend for pre- and post-change points.

Station	Pre-change point	Post-change point	Station	Pre-change point	Post-change point
Dhaka	− 1.02	− 1.82	Barisal	− 1.2	− 1.4
Mymensingh	− 1.5	− 2.3	Bhola	− 1.7	− 1.9
Faridpur	− 2.2	− 2.05	Khepupara	1.25	1.75
Madaripur	− 2.75	− 2.51	Patuakhali	− 1.7	− 1.65
Srimangal	0.12	0.15	Chandpur	− 1.5	0.15
Sylhet	− 1.92	− 1.76	Teknaf	0.5	0.82
Bogra	− 1.95	− 2.22	Chittagong	0	− 0.15
Rajshahi	0.65	0.45	Comilla	− 1.2	0.54
Ishurdi	− 1.3	− 1.5	Cox's Bazar	− .75	− 1.03
Dinajpur	− 3.01	− 3.81	Feni	− 1.3	− 1.01
Rangpur	− 2.3	− 2.6	M.Court	− 3	− 1.5
Jessore	− 1.1	− 1.2	Rangamati	0.75	0.22
Khulna	0.15	0.21	Sandwip	1	1.55
Satkhira	− 0.41	0.75	Sitakunda	0.15	0.21

**Figure 10:**
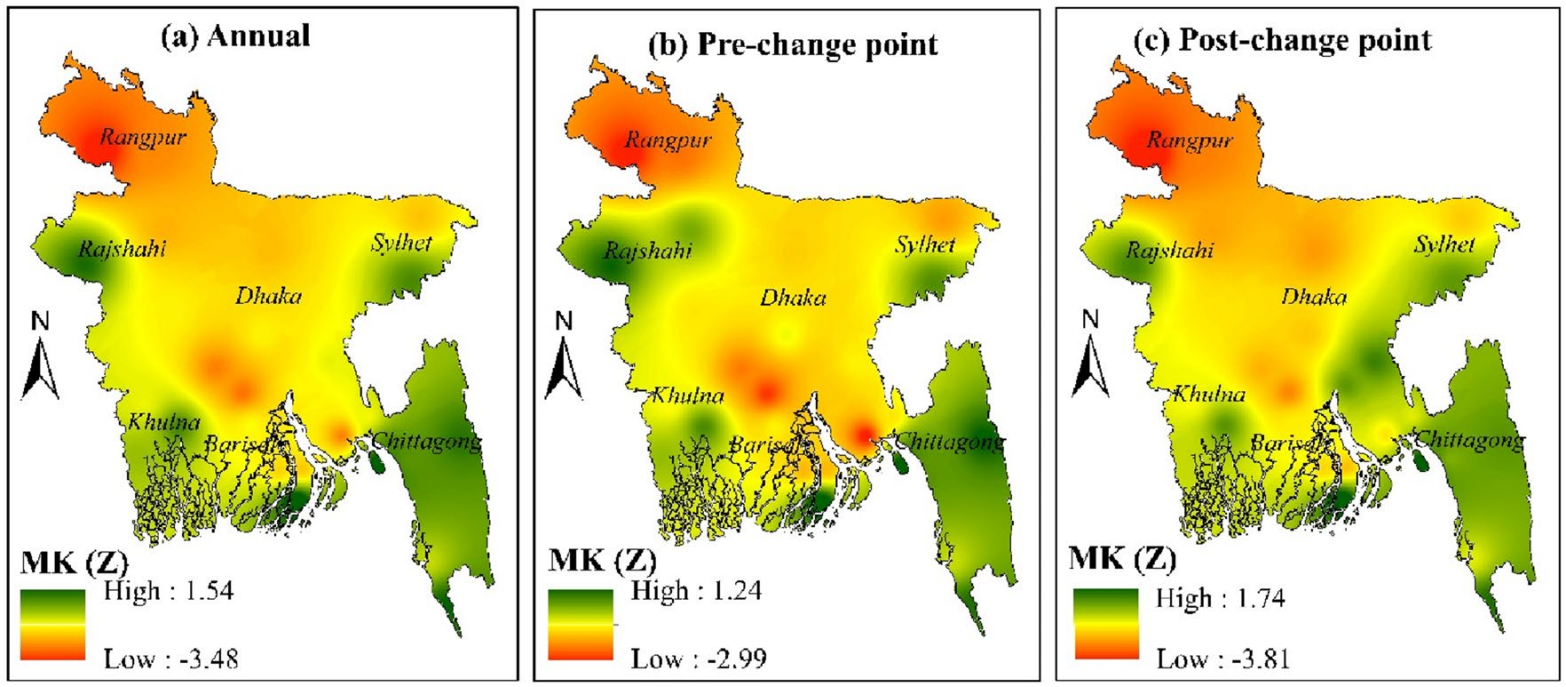
Spatial trend in annual rainfall for pre and post-duration of change point: (**a**) annual rainfall trend, (**b**) rainfall trend in pre-change point, and (**c**) rainfall trend in post-change point, prepared by the authors using ArcGIS software version 10.5, (https://www.esri.com/en-us/arcgis/products).

There was a significant negative trend observed in the post-change point than the pre-change point. Rainfall declined after the change point in the northern and northwestern regions and raised in the center to the southeastern part of Bangladesh (Fig. 10).

#### Slope in innovation trend

**Figure 11:**
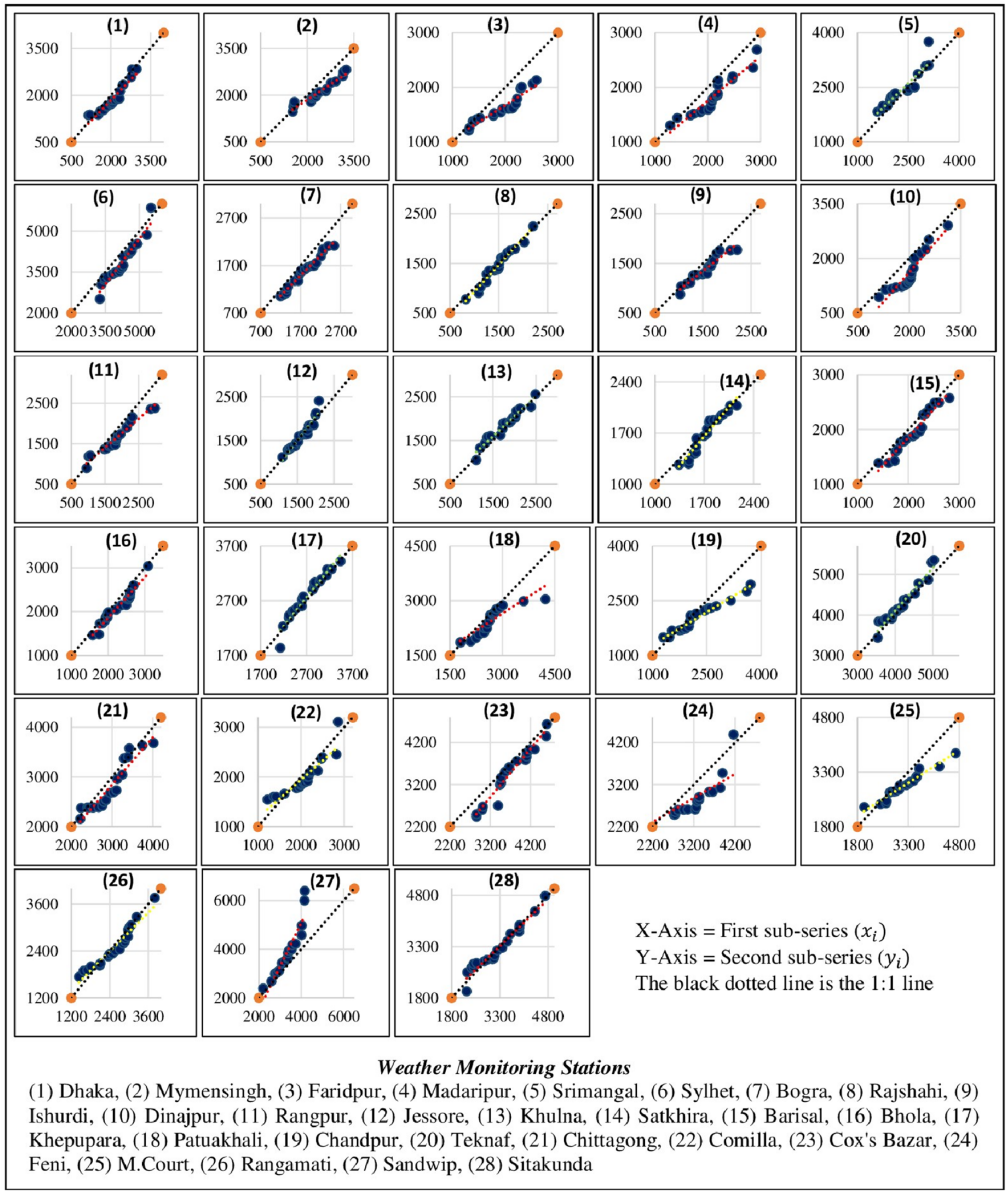
Weather station-wise innovative trend in annual rainfall.

The innovation trend in annual rainfall observed that there are 86% of weather stations has a declining slope and 14% have a rising slope. Figure 11 shows the weather station-wise innovative trend in annual rainfall.

### Predicting rainfall in Bangladesh

**Figure 12:**
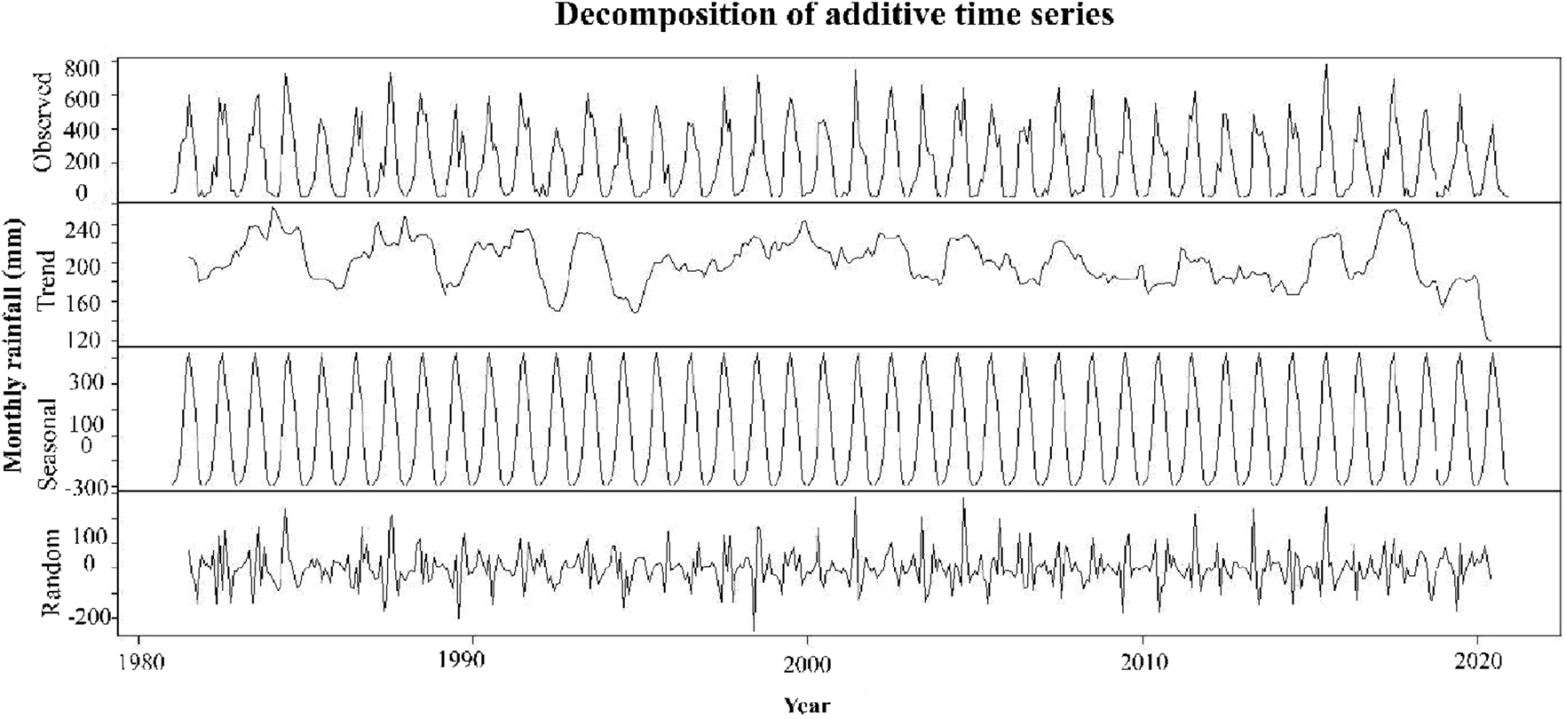
Decomposition of additive time series of monthly rainfall data from 1981 to 2020.

The traditional predicting method in meteorology, the MLP neural network model is used to predict overall monthly and seasonal rainfall for Bangladesh. Single data set for Bangladesh’s rainfall is produced from the observed 28 station data. Figure 12 shows the decomposition of monthly rainfall data from 1981 to 2020 in an additive time series. The observed values are displayed at the top of this decomposition, while the randomly selected data set is displayed at the bottom. The center of this additive time series decomposition shows the seasonal pattern of this data collection and its tendencies (Fig. 12).

**Figure 13:**
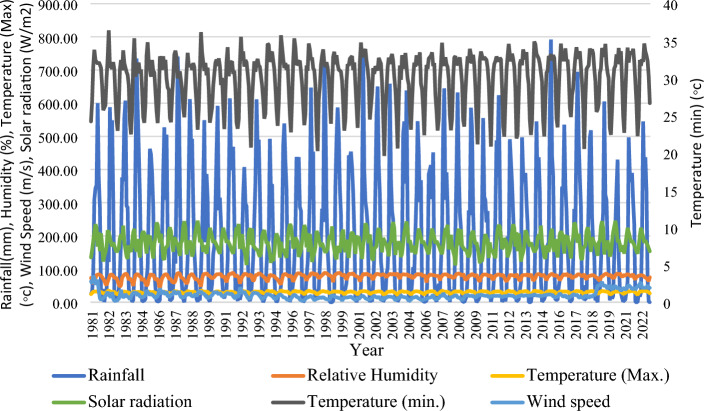
Time series plot of rainfall indicators in this prediction model.

The time series plot of different rainfall indicators is shown in Fig. 13. The time series of maximum and minimum temperature, relative humidity, solar radiation, and wind speed are shown along with rainfall in Fig. 13.

RMSE and correlation (R), two fundamental scoring measures, are used to assess the suggested model. Using a testing dataset, we assess the proposed model's prediction abilities, and the results are shown in Table 6 below. The results show that the suggested model works well since it reduces all kinds of mistakes.

**Table 6:** Evaluation of the performance of the proposed model.

Predictive model	Evaluation metrics					
	RMSE			R		
	Training	Testing	Validation	Training	Testing	Validation
MLP	10.2	11.3	10.8	0.87	0.83	0.85

**Figure 14:**
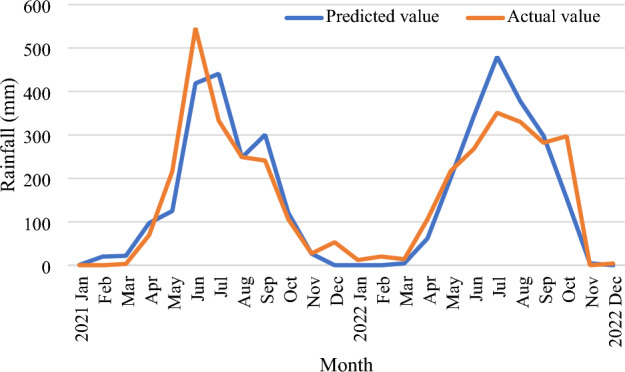
Evaluation of the model by testing set for 24 months.

The outcomes of the proposed MLP-based rainfall prediction model for forecasting rainfall for the ensuing 24 months are shown in Fig. 14. The outcomes demonstrate that the suggested technique has an accuracy of more than 90% for estimating average rainfall (in mm). On the graph, the blue line shows the amount of average rainfall predicted by the suggested model, while the brown line reflects the actual amount of average rainfall. The suggested model may thus be used to forecast rainfall over the next eight years (till 2030).

**Figure 15:**
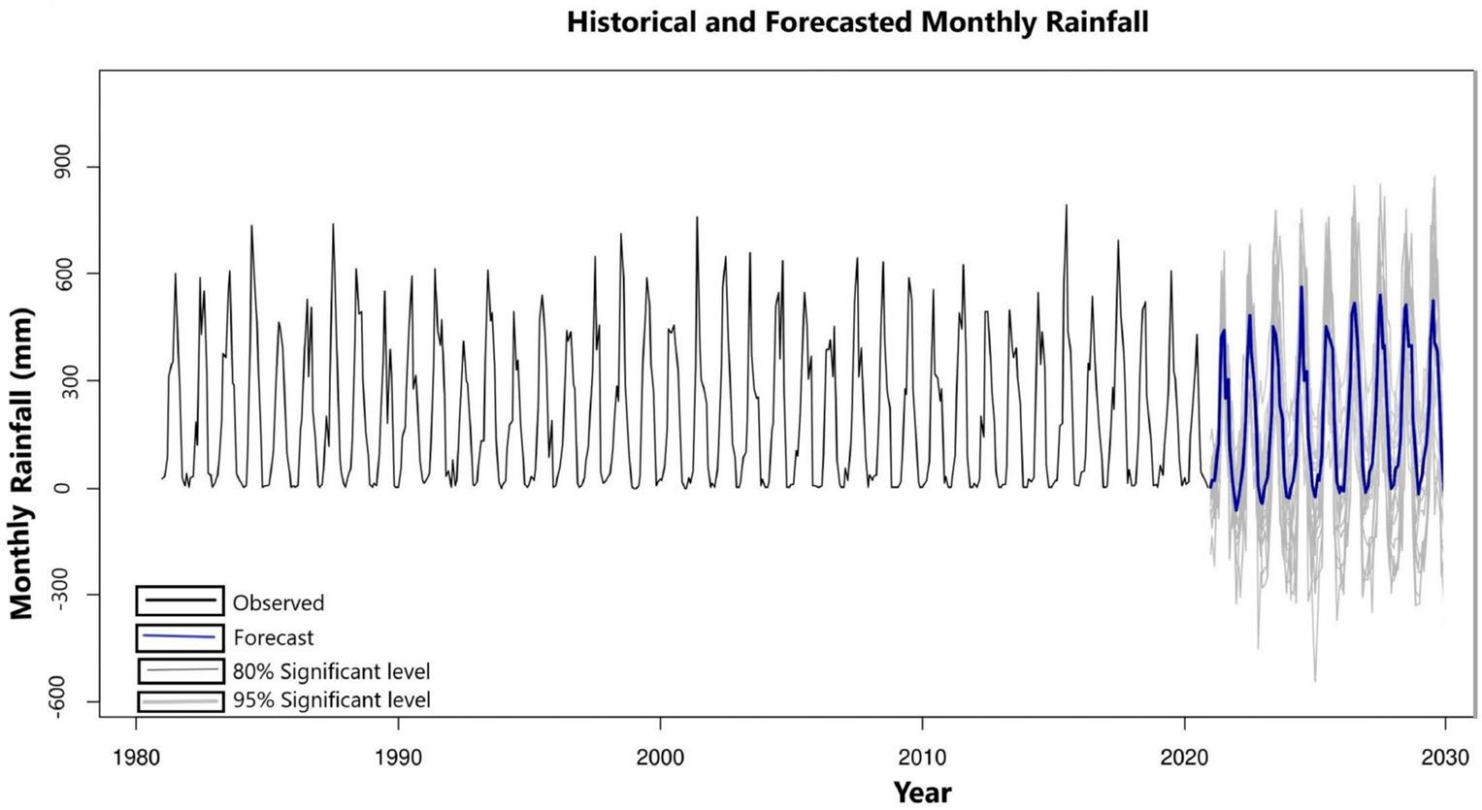
Predicted monthly rainfall for Bangladesh to 2030.

The predicted monthly rainfall till 2030 with 95% confidence levels using the MLP technique is shown in Fig. 15. There was a very minimal error in predicted rainfall, the RMSE was less than 12 mm.

Though this predicted data set contained rainfall below the baseline (< 0 mm) for a few months, we counted it as no rainfall (0 mm). The predicted monthly rainfall is presented in Table 7.

**Table 7:** The range of predicted monthly rainfall in Bangladesh till 2030 (95% confidence level).

Year	Month					
	Jan	Feb	Mar	Apr	May	Jun
2023	0	4.1–5.4	26.5–30.1	90.1–92.3	199.2–220.2	428.1–473.1
2024	0	0	20.1–23.4	89.1–92.5	229.4–253.6	375.6–415.2
2025	0	35.2–38.7	20.1–22.6	90.3–94.5	222.0–245.4	427.8–472.8
2026	0.8–0.95	0	45.4–49.8	165.4–174.5	289.0–319.4	459.1–507.5
2027	4.9–5.3	40.2–45.6	63.4–70.1	109.8–113.4	224.3–247.9	475.7–523.5
2028	7.2–8.1	50.1–53.4	60.1–65.4	135.3–141.2	274.7–303.7	473.7–523.5
2029	6.1–7.4	37.6–41.2	78.9–82.3	121.2–127.8	225.2–248.9	396.2–437.9
2030	0	19.3–24.3	57.8–61.2	95.4–100.1	166.4–184.0	456.3–504.3

Year	Month					
	Jul	Aug	Sep	Oct	Nov	Dec
2023	403.6–446	324.6–359.8	218.9–241.9	182.7–201.9	37.1–41	0
2024	532.7–588.7	283.9–313.7	308.4–340.8	132.0–145.8	55.6–61.4	6.6–7.2
2025	403.0–445.4	391.3–432.5	365.4–403.8	194.4–214.8	16.5–18.3	0
2026	488.5–539.9	399.3–441.3	320.9–354.7	172.4–190.6	59.9–66.2	0
2027	510.2–563.9	369.1–407.9	377.6–417.4	202.9–224.3	43.0–47.6	0
2028	484.5–535.5	372.8–412	379.1–419	185.0–204.4	85.2–94.3	0
2029	498.2–550.6	383.3–423.7	363.4–401.6	202.6–224	103.2–114	0
2030	477.9–528.3	331.5–366.3	358.2–396	194.7–215.1	0	0

A significant fluctuation in predicted monthly rainfall is observed from 2021 to 2030. There will be no rain in December and January. It will not rain for a significant number of years for November and February. So, Bangladesh will suffer water scarcity during dry periods till 2030 (November to February). Predicted maximum rainfall for March, April, May, June, July, August, September, and October are observed in 2029, 2026, 2026, 2027, 2027, 2026, 2027, and 2028. The predicted result indicates that there will be much higher rainfall from 2026 to 2029 (Table 7). On the other hand, predicted minimum rainfall for March, April, May, June, July, August, September, and October are observed respectively in 2024, 2024, 2030, 2024, 2025, 2024, 2023, and 2026 (Table 7). The predicted result suggests that comparatively lower rainfall will occur in the first and last quarter of the study period.

The predicted monthly rainfall, converted into seasonal rainfall, is presented in Table 8.

**Table 8:** Bangladesh’s predicted average seasonal rainfall till 2030.

Year	Seasonal rainfall (mm)		
	Pre-monsoon	Monsoon	Post-monsoon
2023	315.8–342.6	1557.2–1772.7	41.2–46.4
2024	338.6–369.5	632.6–1804.2	62.2–68.6
2025	332.4–362.5	1781.9–1969.3	51.7–57
2026	499.8–543.7	1840.2–2034	60.7–67.15
2027	397.5–431.4	1935.5–2137	88.1–98.5
2028	470.1–510.3	1895.1–2094.4	142.5–155.8
2029	425.3–459	1843.7–2037.8	146.9–162.6
2030	319.6–345.3	1818.6–2010	19.3–24.3

The maximum predicted rainfall for pre-monsoon, monsoon, and post-monsoon seasons is observed in 2028, 2027, and 2029. On the other hand, the lowest rainfall for these seasons is observed in 2023, 2023, and 2030 respectively.

### Reasons for rainfall change

**Figure 16:**
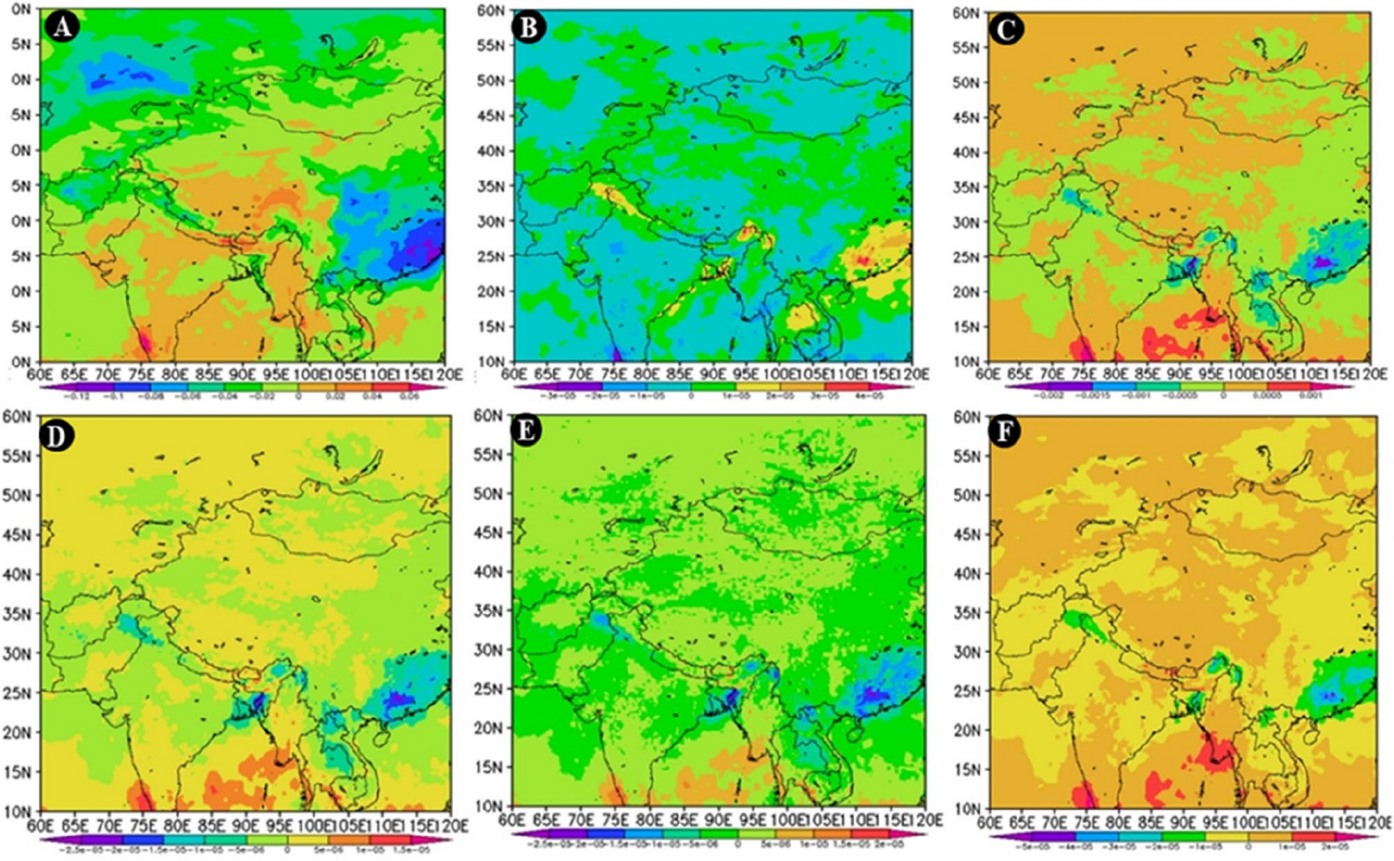
Spatial variations of differences (**A**) Low cloud Cover, (**B**) Mean vertically Integrated Moisture Divergence, (**C**) Convective Precipitation in monsoon season, (**D**) Mean convective precipitation rate, (**E**) Convective rainfall rate in post-monsoon season, (**F**) Mean total precipitation rate between the recent phase of 2002–2020 and 1980–2001, prepared by the authors using GrADS version **2.2.1 (**http://cola.gmu.edu/grads/downloads.php).

Most areas of Bangladesh experience moderate to heavy convective precipitation; however, when convective rainfall rates were low, more water vapor from the Bay of Bengal was transported to the land surface, which is unfavorable for the production of raindrops (Fig. 16). The mid-central and northeastern parts of the nation saw a drop in mean rainfall during the monsoon season as a result (Fig. 16). The country covers a trivial region with elevated geopotential height change and transports fewer moisture vapors from the Bay of Bengal to the local land masses, responsible for rainfall change^44^. This is the reason for an increase in yearly rainfall in summer monsoons noticed in the country's northeastern and southeastern parts^86^. Throughout the nation, the southeasterly breeze was intensified, reducing the intrusions of colder air and decreasing rainfall throughout Bangladesh in the post-monsoon season. The development of the anticyclonic circulation reduces the power of the southeast wind, which in turn strengthen the summer monsoon season and rise warmer air into Bangladesh and ultimately causes an increase in rainfall^138^. Contrarily, the majority of the areas saw moderate to low mean convective precipitation rates, which to some degree reduced rainfall^147^. Overall, the number of low clouds has grown throughout the country and neighbor regions^27^. The few cloud coverings will boost the atmosphere's condensing effect on surface solar radiation, which will reduce the trend in rainfall^124^. The country as a whole saw a high mean total precipitation rate, except for the west, which caused an inconsistent downdraft pattern and increased the number of clear sky days over the most recent research period (1980–2020). The vast majority of Bangladesh's regions showed a lessening vertically integrated moisture divergence that was brought on by a considerable decrease in rainfall.

## Discussion

Evaluation of historical rainfall trends is crucial in many fields, including water resource management, planning for sustainable agriculture, managing ecosystems, and the health sector^52^. The variability of rainfall trends on monthly and seasonally was thoroughly investigated using the rainfall data from 40 years from 28 observation stations. The statistical analysis reveals that from 1981 to 2020, the highest average annual rainfall was observed in Teknaf (4212.9 mm) and the lowest in Ishurdi (1466.4 mm). From 1958 to 2007, the mean annual rainfall in Bangladesh was 1527 mm to 4193 mm, according to Shahid^62^. For monthly rainfall, the maximum average rainfall recorded in July was 461.9 mm (21.7% of annual rainfall). 57.6% rainfall was observed from July to August. However, 50% of rainfall was recorded from 1958 to 2007^39^. Compared with much older studies, rainfall during the dry season decreased from the 1950s to the 2010s while monsoon rainfall increased^43,49,60,123,124^. This fluctuation in rainfall distribution over a year significantly affects changing climatic conditions and agricultural production. The World Bank^125^ observed it at 2200 mm in the last 30 years, though the present study followed the average annual rainfall in Bangladesh at 2432.6 mm in the previous 40 years. Yousuf and Ahmed^48^ also observed average annual rainfall in Bangladesh (1948–2011) was 2456.38 mm. It is obvious that from the 1940s to the present, Bangladesh's yearly rainfall average gradually dropped. Throughout the study period, the average rainfall varied from 279 to 1076 mm before the monsoon, 1133 to 3754 mm during the monsoon, and 40 to 120 mm after the monsoon. Minimal different results were observed by the World Bank^125^ from 1991 to 2020. Rainfall was found to vary in Bangladesh; Islam et al.^49^ observed that most of the annual rainfall in Bangladesh was recorded in the southern and eastern parts. The southern area has the most significant value of the precipitation concentration index (LCI)^44^. Shahid and Khairulmaini^124^ also predict that the southern part will remain slightly subject to normal rainfall conditions. Similarly, the rainfall is higher in the southeast, according to the present study, though it is lower in northwest Bangladesh.

According to the MK Z value, over 80% of monitoring stations face a declining trend for January, February, March, August, November, and December. April, May, June, and September observed 60% to 80% monitoring stations for a declining trend. Almost 77% of stations have a declining tendency, and 23% have a rising trend for monthly rainfall. No previous comprehensive studies focus on the monthly rainfall trend in Bangladesh. In Indian Sub-Continent, Praveen et al.^27^ found a similar result, a significant negative trend for overall annual rainfall. In this current study, the month of May–July have a mixed pattern of rising and declining trends. Rahman et al.^46^ observed a mixed pattern of Long-term monthly negative and positive trends from February to September. According to this study, the number of monitoring stations has a marked dropping tendency throughout the pre-monsoon, monsoon, and post-monsoon periods, respectively, 82%, 75%, and 100%. Shahid and Khairulmaini^124^ also note a falling trend in the precipitation concentration at most locations. Endo et al.^40^ also found that the frequency of days with light precipitation rises throughout the pre-monsoon season. Shahid^39^, Endo et al.^40^, and Zannat et al.^41^ showed a changing pattern in monsoon rainfall in Bangladesh, and it was increasingly reported by Sing^126^. Spatiotemporal variation in monsoon rainfall in Bangladesh is also affected by EI-Nino/Southern Oscillation (ENSO)^127,128^. The findings from far older studies that looked at rainfall trends in Bangladesh between the 1950s and the 2000s are shown different results^129–131^. Those studies found significant positive trends at most weather stations during the post-monsoon and pre-monsoon^31,42,43,60,132^. However, this study observed that all weather stations have a declining trend in post-monsoon rainfall^146^. Mullick et al.^57^ observed that the average rainfall indicates a rising tendency in Bangladesh, except in the winter. However, this present study found only 18% of weather stations in pre-monsoon and 25% of weather stations in the monsoon season, which has a rising trend in rainfall. The trend in annual rainfall is significantly declining after the change point. According to an innovative trend test, 86% of monitoring stations have a declining tendency. Only 14% of stations have a rising trend. The overall trend of rainfall, especially the post-monsoon rainfall in Bangladesh, is declining.

The majority of previous research on rainfall predicting has used Linear Regression, Adaptive Neuro-Fuzzy Inference System (ANFIS), Genetic Algorithm (GA), Mann Kendall test, Deep Learning Approach, Feed Forward Neural Network (FFNN), and Empirical and Dynamical Methods^10,11,68,70^. These methods never predict the actual scenario of future rainfall. We used the Multilayer perception neural network technique to predict the rainfall not only seasonal scale but also monthly rainfall. Banik et al.^11^ used the ANN method to predict rainfall, but only during the monsoon season in Bangladesh. A significant fluctuation in predicted monthly rainfall is observed from 2021 to 2030. The maximum predicted rainfall for pre-monsoon, monsoon, and post-monsoon seasons is observed in 2028, 2027, and 2029.

On the other hand, the lowest rainfall for pre-monsoon, monsoon, and post-monsoon seasons will occur in 2023, 2023, and 2030 respectively. Only some studies concluded that there would be no significant trends in Bangladesh^124^, which shows an irregular pattern in rainfall trends in Bangladesh^49,72^. In the present study, the predicted results also show anirregular pattern in future rainfall in Bangladesh. Also, Praveen et al.^27^ predict the upcoming 15 years of rainfall over India in 2020, exhibiting a significant decline in rainfall. An assessment of long-term water availability at various spatiotemporal weighting scales is required as the likelihood of adverse effects of changing climate on water resources rises^148^. To mitigate the potential impacts of climate change, this research is anticipated to provide insight into the management and development of Bangladesh's agricultural and water resources.

Statistical findings indicate that most rainfall occurs in the monsoon season, of which more than half of annual rainfall occurs from June to August. The pre-monsoon and post-monsoon have very little rainfall. Rainfall is become higher during the monsoon and becomes lower during the post-monsoon. According to a trend test, Bangladesh's rainfall decreases during the post-monsoon season overall while increasing in some areas during the pre-monsoon. According to this phenomenon, Monsson rains appear to occur earlier than expected in a calendar year. Identical changes in monsoon rainfall patterns were also observed in neighboring countries^16,86,133^. This fluctuation is evident in the changing rainfall pattern over the year, promoting the meteorological drought^134^. Bangladesh's southern and eastern parts observed healthy rainfall across all seasons, while less rainfall was observed in the western part. Very little rainfall was observed in northern Bangladesh during the post-monsoon. If this process continues in the northern and western parts of Bangladesh, these areas will become drought in recent future. However, groundwater drought already affects northwestern Bangladesh^106,135,136^. The trend test for monthly rainfall shows that more than 80% station's rainfall declined from November to March and August. Contrary, only the month of July and October have a rising trend for more than 50% of monitoring stations. Bangladesh has a declining trend in rainfall during the post-monsoon; only some areas of northern and southern parts have a rising trend for the pre-monsoon and monsoon, respectively. The post-monsoon rainfall declined over Bangladesh, while pre-monsoon and monsoon rainfall rose for some areas. The predicted result shows the fluctuating rainfall pattern in the upcoming years. Bangladesh's weather sites offer a declining tendency during post-monsoon, and the predicted rainfall fluctuates, which is strong evidence of climate change. Any shortage in rainfall may impact agriculture and groundwater resources. Because agricultural practice depends on water supply, it comes from rainfall or underground sources. Although, the long-term changes in the rainfall pattern are primarily associated with changes in sea surface temperature (SST) and land-surface processes. The atmospheric process is more of a response to the changes in the SST and other slowly varying parameters. This is one of the main limitations of this study and deserves further investigation.

## Conclusion

This study uses a monthly and seasonal rainfall dataset to investigate Bangladesh's historical rainfall pattern and projection. The Mann–Kendall, innovative trend analysis, and Sen's Slope Estimator were performed to detect trends in historical rainfall in Bangladesh. A multilayer perception neural network was used to predict future rainfall patterns, and the causes of rainfall changes are also explored in the present study. The following key conclusions are drawn from the current study:

- Rainfall has a declining trend for the post-monsoon season all over Bangladesh, and it also declines for more than 75% of areas for the other two seasons (pre-monsoon and monsoon). Above 80% of monitoring stations have a declining trend from November to March, particularly August, while more than 50% have a rising trend during July and October. This fluctuation indicates a substantial change in rainfall patterns, especially monsoon rainfall.
- According to the predictions, future rainfall in Bangladesh will likely follow an erratic pattern. During the prediction period (till 2030), there will be no rain in December and January.
- An increasing/decreasing convective precipitation rate, enhanced low cloud cover, and insufficient moisture divergence in the Bay of Bengal being transported to the northwest direction may have greatly influenced changes in rainfall in Bangladesh, according to large-scale ocean-atmospheric changes derived from the ECMWF ERA5 reanalysis data
- This study encountered missing data for several locations across several years, impacting the trend analysis because estimated data can only partially replace actual data. However, further studies are necessary to identify the factors behind the rainfall pattern and will use earth and atmospheric factors with rainfall data to predict rainfall.
- This study's findings are predicted to impact Bangladesh's water resource management and agricultural planning significantly. Cropping types and patterns that have been developed may need to respond to variations in rainfall patterns.

## Data Availability

The datasets generated and/or analyzed during the current study are not publicly available due to restrictions imposed by the BMD authority but are available from the corresponding author upon reasonable request.
